# Chromosome‐scale genome assembly‐assisted identification of *Mi‐9* gene in *Solanum arcanum* accession LA2157, conferring heat‐stable resistance to *Meloidogyne incognita*

**DOI:** 10.1111/pbi.14055

**Published:** 2023-04-19

**Authors:** Lijun Jiang, Jian Ling, Jianlong Zhao, Yu Yang, Yuhong Yang, Yan Li, Yang Jiao, Zhenchuan Mao, Yunsheng Wang, Bingyan Xie

**Affiliations:** ^1^ State Key Laboratory of Vegetable Biobreeding Institute of Vegetables and Flowers, Chinese Academy of Agricultural Sciences Beijing China; ^2^ Institute of Vegetables, Shandong Academy of Agricultural Sciences Jinan Shandong China; ^3^ Institute of Modern Agriculture on Yellow River Delta, Shandong Academy of Agricultural Sciences Jinan Shandong China; ^4^ College of Plant Protection Hunan Agricultural University Changsha Hunan China

**Keywords:** *Solanum arcanum* LA2157, *Mi‐9*, genome assembly, Hi‐C, VIGS, tomato genetic transformation

## Abstract

Root‐knot nematodes (RKNs) are infamous plant pathogens in tomato production, causing considerable losses in agriculture worldwide. *Mi‐1* is the only commercially available RKN‐resistance gene; however, the resistance is inactivated when the soil temperature is over 28 °C. *Mi‐9* in wild tomato (*Solanum arcanum* LA2157) has stable resistance to RKNs under high temperature but has not been cloned and applied. In this study, a chromosome‐scale genome assembly of *S. arcanum* LA2157 was constructed through Nanopore and Hi‐C sequencing. Based on molecular markers of *Mi‐9* and comparative genomic analysis, the localization region and candidate *Mi‐9* genes cluster consisting of seven nucleotide‐binding sites and leucine‐rich repeat (NBS‐LRR) genes were located. Transcriptional expression profiles confirmed that five of the seven candidate genes were expressed in root tissue. Moreover, virus‐induced gene silencing of the *Sarc_034200* gene resulted in increased susceptibility of *S. arcanum* LA2157 to *Meloidogyne incognita*, and genetic transformation of the *Sarc_034200* gene in susceptible *Solanum pimpinellifolium* conferred significant resistance to *M. incognita* at 25 °C and 30 °C and showed hypersensitive responses at nematode infection sites. This suggested that *Sarc_034200* is the *Mi‐9* gene. In summary, we cloned, confirmed and applied the heat‐stable RKN‐resistance gene *Mi‐9*, which is of great significance to tomato breeding for nematode resistance.

## Introduction

Tomato (*Solanum lycopersicum*) is native to South America and is one of the most essential vegetable crops with a top annual production in the world (Bai and Lindhout, 2007; Peralta and spooner, 2005). Long‐term domestication and continuous selection for desirable traits make cultivated tomatoes more susceptible to various stresses. Root‐knot nematodes (RKNs, i.e., *Meloidogyne* spp.) are small soil‐borne biotrophic parasites with a wide host range of more than 2000 species which pose a serious threat to tomato production, and are listed as the most dangerous plant pathogenic nematodes (Castagnone‐Sereno *et al*., 2013; Jones *et al*., 2013; Trudgill and Blok, 2001). The most typical characteristic of infected plants is the appearance of galls or root knots, leading to symptoms such as stunted growth, wilting, reduced fruit yield and increased susceptibility to infections from other pathogens (Williamson, 1998). Although nematicides have been successfully used in the agricultural production, considering the high cost and toxicity in the environment, utilizing of host resistance can be regarded as an effective and sustainable alternative control measure (Devran *et al*., 2010; Devran and Elekçioğlu, 2004; Oka *et al*., 2000; Ploeg, 2002; Roberts and Thomason, 1986).

Tomato resistance to RKNs was first identified in *Lycopersicon peruvianum* (Mill.P.I.128657), and subsequently shown to be encoded by a single dominant gene named *Meloidogyne Incognita‐1* (*Mi‐1*) (Bailey, 1941). There are seven homologues of *Mi‐1*, of which only *Mi‐1.2* is resistant to nematodes (Milligan *et al*., 1998; Rossi *et al*., 1998). *Mi‐1* was transferred from *L. peruvianum* to the cultivated tomato *Lycopersicon esculentum* by embryo rescue and traditional interspecific hybridization and has been a commercially viable source of RKN resistance in tomatoes for over 60 years (Medina Filho and Stevens, 1980; Smith, 1944). In subsequent studies, *Mi‐1* was cloned by positional cloning and belonged to the resistance gene containing leucine zipper (LZ), nucleotide binding site (NBS) and leucine‐rich repeat region (LRR) domains (Milligan *et al*., 1998; Vos *et al*., 1998). *Mi‐1* can effectively resist *M*. *incognita*, *Meloidogyne javanica* and *Meloidogyne arenaria*, and has been widely used to control RKNs in tomato production; however, the resistance is inactivated when the soil temperature is over 28 °C (Dropkin, 1969; Holtzmann, 1965; Williamson, 1998).

In the context of global warming, the exploration and application of high‐temperature stable nematodes‐resistant germplasm resources are of great development prospects. To date, 10 RKN‐resistant tomato genes have been reported. Among them, only five genes had been mapped, and seven genes (including *Mi‐2*, *Mi‐3*, *Mi‐4*, *Mi‐5*, *Mi‐6*, *Mi‐9* and *Mi‐HT*) showed resistance to RKNs under high temperatures (El‐Sappah *et al*., 2019; Wu *et al*., 2009). However, these heat‐stable resistance genes have not been successfully transferred to cultivated tomatoes and used in agriculture (Veremis *et al*., 1999). LA2157 is an accession belonging to the ancient Maranon race complex of *S. peruvianum* from the Maranon drainage area in northern Peru, and was later assigned to *S. arcanum*, which is a new wild tomato species segregated from *S. peruvianum* sensu lato (Peralta *et al*., 2005; Veremis *et al*., 1999). A previous study showed that the heat‐stable resistance mediated by *S. arcanum* LA2157 to RKNs is mediated by *Mi‐9*, i.e., a single dominant resistance gene (Veremis *et al*., 1999). *S. arcanum* and cultivated tomatoes are two different *Solanum* species, and unlike most *S. arcanum* accessions, LA2157 is self‐compatible (Jablonska *et al*., 2007). *Mi‐9* is a homologue of *Mi‐1*, which is located between two markers (C32.1 and C8B) on the short arm of chromosome 6 (Jablonska *et al*., 2007). Six markers are currently available to locate *Mi‐9*; two are based on Restriction Fragment Length Polymorphism (RFLP) (C32.1 and C264.2) and four are based on Polymerase Chain Reaction (PCR) (CT119, REX‐1, *Aps‐1* and C8B) (Ammiraju *et al*., 2003; Jablonska *et al*., 2007). *Mi‐9* showed resistance to *M*. *incognita*, *M*. *javanica* and *M*. *arenaria*, and exhibited excellent stable resistance to RKNs under a high temperature (>28 °C) (Ammiraju *et al*., 2003; El‐Sappah *et al*., 2019; Jablonska *et al*., 2007; Veremis *et al*., 1999). Determining the way in which to successfully clone *Mi‐9* and introduce it into the cultivated tomato plants would provide a valuable genetic improvement for tomato production with good environmental adaptability under potential climate impacts of global warming.

Positional cloning is a common and classical method for cloning plant disease resistance genes; the first gene to be obtained using this method was the *Pto* gene (Martin *et al*., 1993). Even in the absence of advanced knowledge of the sequence or gene product, knowledge of sufficient molecular markers and hybrid populations would enable accurate identification of the major genes that control traits; however, this process is costly and inefficient when the plant material genome is large and has many repeated sequences. Many advanced methods for cloning resistance genes have been reported in recent years. The pan‐genome variation of wild diploid wheat was exploited through *k*‐mer‐based association genetics with *R* gene enrichment sequencing (AgRenSeq), and four stem rust resistance genes were cloned (Arora *et al*., 2019). MuRenseq (i.e., a three‐step method combining chemical mutagenesis, exon capture and sequencing for cloning of the *R* gene) has previously been described, and stem rust resistance genes *Sr22* and *Sr45* were cloned from hexaploid bread wheat using this method (Steuernagel *et al*., 2016). Furthermore, it has been reported that *Rpi‐amr3i* was cloned by combining RenSeq and single‐molecule real‐time (SMRT) (Witek *et al*., 2016). In addition, an extreme resistance (ER) gene *Ry*_*sto*_ of potato virus Y (PVY) was isolated using the same method that combined RenSeq and SMRT (Grech‐Baran *et al*., 2020). However, many of these methods may be cumbersome; for example, mutational genomics may take numerous generations to screen thousands of mutant lines. With the rapid development of genome sequencing technology, it is possible to sequence and assemble a whole genome at the chromosome level through third‐generation and Hi‐C sequencing (Li *et al*., 2019; Ling *et al*., 2021; Zhang *et al*., 2019). Combining gene mapping and molecular markers, this represents a cost‐effective method to rapidly identify the candidate resistance genes.

The genome of the inbred tomato cultivar ‘Heinz 1706’ has been sequenced and assembled, with a size of 760 megabases (Mb) assembled in 91 scaffolds aligned to the 12 tomato chromosomes (TomatoGenomeConsortium, 2012). The genome of *Solanum pimpinellifolium* LA1589 was also sequenced and assembled, yielding a draft genome of 739 Mb (TomatoGenomeConsortium, 2012). Other tomato genomes were sequenced and reported in subsequent studies (Bolger *et al*., 2014; Razali *et al*., 2017; Hosmani *et al*., 2019; Wang *et al*., 2020). All these genome sequencing and chromosome assembly data in tomato plants provide favourable support for the discovery of valuable genes based on genomic big data and comparative genomic analysis. In the present study, we sequenced and assembled the genome of *S. arcanum* LA2157 through Oxford Nanopore Technologies (ONT) long reads combined with Illumina short reads and Hi‐C chromatin contact information, then identified the *Mi‐9* genes cluster by combining the reported molecular markers. After transcriptional expression profiling and functional verification by VIGS, the *Sarc_034200* gene was preliminarily identified as the candidate *Mi‐9* gene. Subsequently, the *Sarc_034200* gene was cloned through bacterial artificial chromosome (BAC) screening and long PCR amplification, and transformed into *S*. *pimpinellifolium* (i.e., a variety susceptible to *M. incognita*), conferring resistance to *M. incognita* under high temperature as a result. Taken together, we cloned *Sarc_034200* and confirmed that it was the *Mi‐9* gene and had stable resistance to RKNs under a high temperature of 30 °C. Our findings are expected to provide a valuable resistance trait for tomato breeding.

## Results

### Chromosome‐scale genome assembly of the *S. arcanum* genome

**Figure 1:**
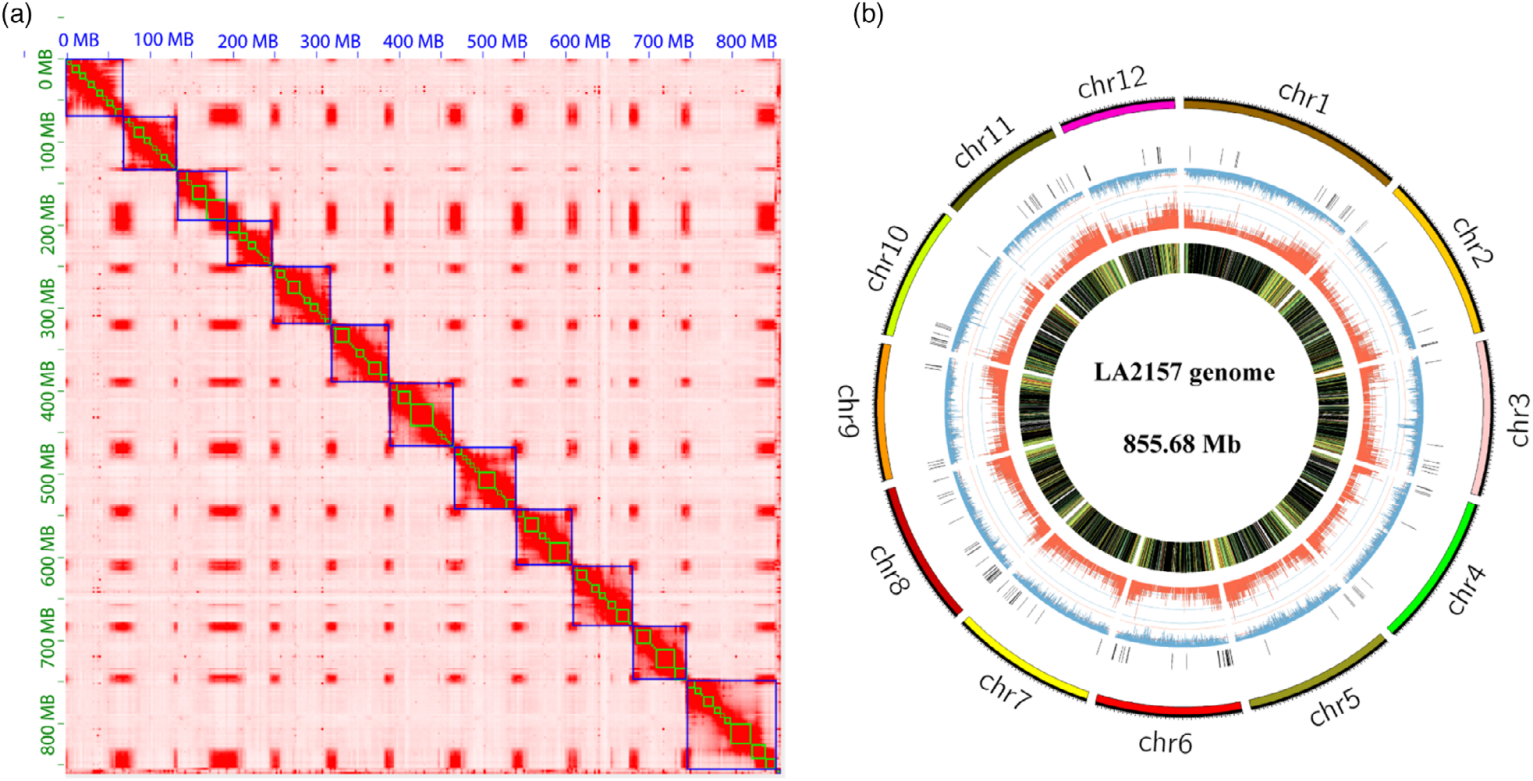
Hi‐C contact map and genome features of LA2157. (a) Hi‐C contact map. Blue blocks indicated the scaffolds (chromosomes). (b) Genome features of LA2157. The outermost circle is the chromosomes. The bar charts from outside to inside in turn are NBS‐LRR genes (black), density of repetitive sequence (blue), gene density (dark red) and gene expression (FPKM).

The genome of LA2157 was assembled using ONT long reads combined with Illumina short reads and Hi‐C chromatin contact information. A total of 83 Gb of ONT reads with an N50 reads length of 33.18 kb was generated, covering approximately 123× of the LA2157 genome with an estimated size of 672.9 Mb (Figure S1). The ONT reads were assembled into contigs, followed by polishing with ONT reads and Illumina reads. The nanopore raw data and final assembled data of LA2157 genome are shown in Table S1. As shown in Table S2, the primary assembly resulted in 144 contigs with a total length of 862.4 Mb and an N50 length of 10.9 Mb. By using Hi‐C data, a total of 855.68 Mb assembly (accounting for 99.2%) was clustered into 12 pseudomolecules (Figure 1a). To assess the assembly completeness, we remapped the pair‐end reads to the assembled LA2157 genome and 98.57% reads could be properly mapped against the genome. A total of 33 489 protein‐coding genes were predicted for LA2157 (Table S2), 23 550 (70.32%) of which were confirmed by RNA‐seq. Among them, 30 833 (92.07%) of the LA2157 genes were functionally annotated (Table S3). Approximately 408.35 Mb (48.73%) of the assembled LA2157 was annotated as repetitive sequences. Among them, 74.34% were retrotransposons (Class I) and 72.58% were long terminal repeats (LTRs) in LA2157 (Figure 1b). BUSCO (Benchmarking Universal Single‐Copy Orthologs) assessment indicated that approximately 98.3% of the core conserved Solanales genes (solanales_odb10) were found complete in the LA2157 assembly. Alignments of the genome of *S. arcanum* LA2157 (*Sa*) with *S. lycopersicum* Heinz 1706 (*Sl*) and *S. pimpinellifolium* LA2093 (*Sp*) showed good collinearity between these genomes (Figure S2). Despite the high collinearity, 264 inversions ranging from 239 bp to 21.9 Mb were identified between LA2157 and Heinz 1706, and 284 inversions ranging from 217 bp to 22.0 Mb were identified between LA2157 and LA2093 (Table S4).

### Identification, chromosome mapping and diversity of NBS‐LRR genes in three tomato species

**Figure 2:**
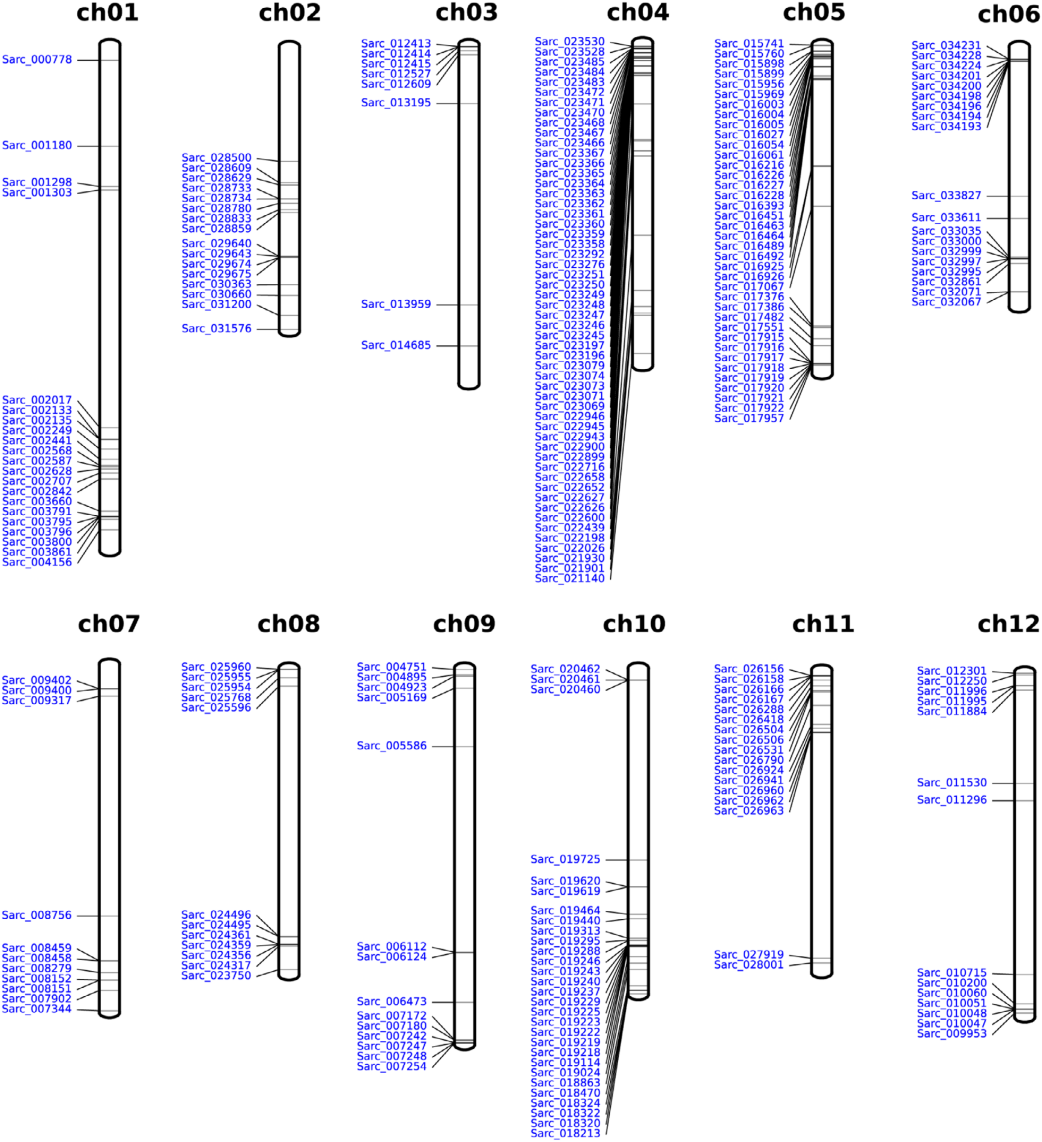
The distribution of NBS‐LRR‐encoding genes of LA2157 on 12 chromosomes.

A total of 253 genes in the LA2157 genome were identified as possible NBS‐LRR genes (Table S5). According to the domain types, the LA2157 NBS‐LRR gene family was classified into six sub‐groups, with 77 N (NBS)‐type, 23 TNL (TIR‐NBS‐LRR)‐type, 4 TN (TIR‐NBS)‐type, 42 CN (CC‐NBS)‐type, 42 NL (NBS‐LRR)‐type and 27 CNL (CC‐NBS‐LRR)‐type (Table S6). LA2157 NBS‐LRR members were unevenly distributed on 12 chromosomes, with the three largest NBS‐LRR gene clusters on chr04 (54 genes), chr05 (38 genes) and chr10 (29 genes), indicating that these chromosomes may undergo large‐scale gene replication events on the genome (Figure 2). We also identified NBS‐LRR genes from two other *Solanum* crop genomes and performed an NBS‐LRR comparative analysis. As shown in Table S6, the genome of LA2157 contained a similar number of NBS‐LRR genes (215) with *S. pimpinellifolium* LA2093 (208) and *S. lycopersicum* (207). Comparative analysis indicated that the number of NBS‐LRR genes in CN type displayed a significant difference (*X* ^2^ = 3.9337, *P* < 0.05) between LA2157 (42) and LA2093 (29), suggesting that the number of this gene type was closely related to the genome variation.

### Gap‐free sequence‐based fine mapping of the *Mi‐9* genes cluster through chromosome 6 comparison of RKN resistant and susceptible tomato species

**Figure 3:**
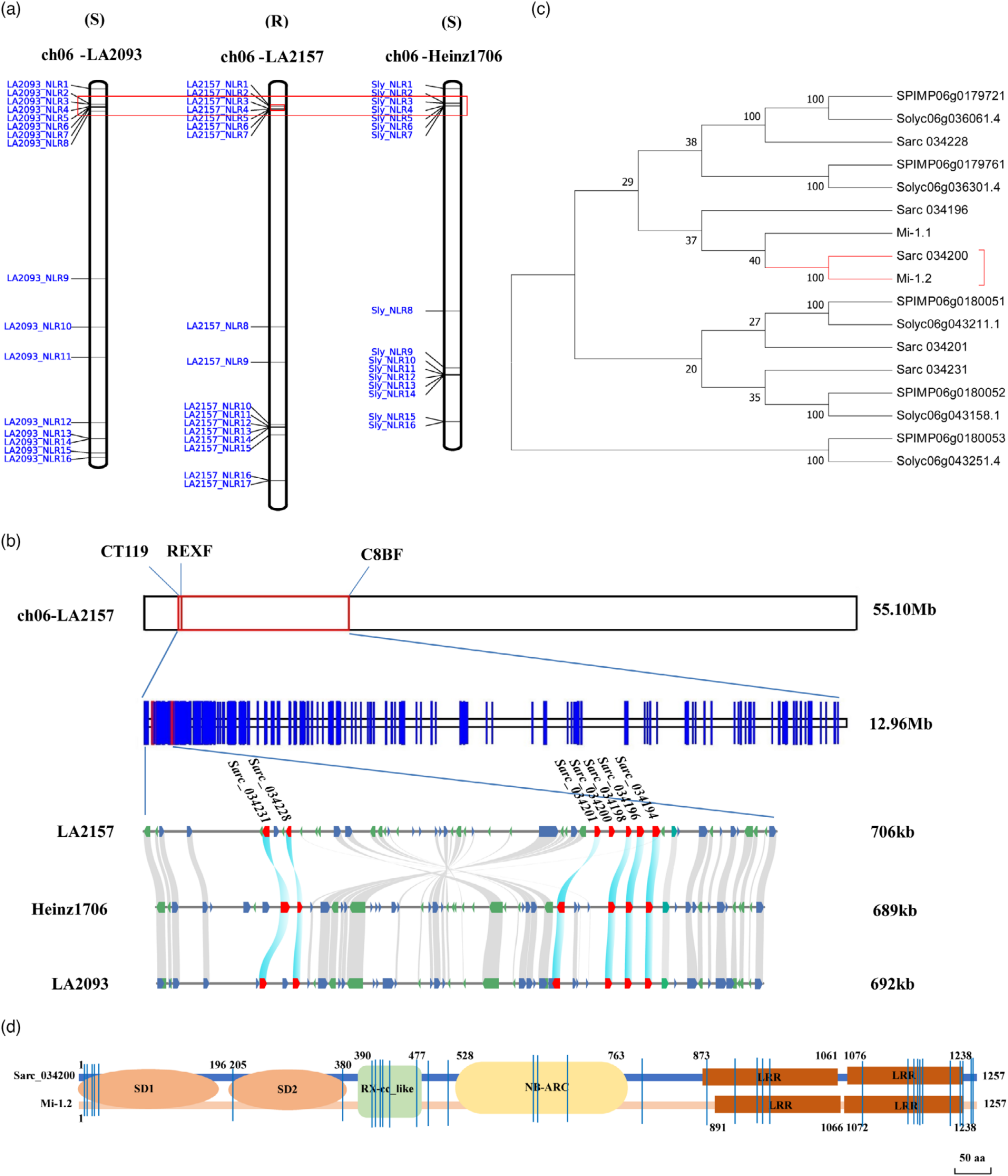
Map of *Mi‐9* genes cluster on chromosome 6 based molecular marker, evolutionary relationship analysis and diagram of amino acids structure comparison between Sarc_034200 and Mi‐1.2. (a) NBS‐LRR genes of *S. arcanum* LA2157, *S. pimpinellifolium* LA2093 and *S. lycopersicum* Heinz 1706 on chromosome 6. The red box indicates the responding regions of *Mi‐9* molecular markers in three species. (b) Molecular markers linked to *Mi‐9* gene and prediction of candidate *Mi‐9* gene. The upper panel shows the molecular makers (CT119, REXF and C8BF) on LA2157 chromosome 6. The black rectangle represents chromosome 6 (ch06‐LA2157, 55.10 Mb in length) and red rectangles represent locations of the molecular markers on ch06‐LA2157. The middle panel shows the 12.96 Mb region between CT119 and C8BF. Blue vertical lines represent genes and red vertical lines represent NBS‐LRR genes in the region. The lower panel shows the 706 kb NBS‐LRR region in the middle panel and the microsynteny analysis of the NBS‐LRR region with *S. lycopersicum* Heinz1706 and *S. pimpinellifolium* LA2093. The chromosome segments (706 kb) are shown as horizontal lines, and arrows represent genes and their orientations. The red arrows represent NBS‐LRR genes. Conserved orthologous gene pairs among LA2157, Heinz1706 and LA2093 are connected with grey lines, and the connection between NBS‐LRR orthologous gene pairs is highlighted by sky‐blue lines. (c) Evolutionary relationship of *Mi‐9* genes cluster, *Mi‐1*, and the genes in the same chromosome segment with *Mi‐9* genes cluster in *S. lycopersicum* Heinz 1706 and *S. pimpinellifolium* LA2093. (d) The amino acid structure comparison between Sarc_034200 and Mi‐1.2. The blue vertical lines represent the positions of non‐synonymous mutations.

The NBS‐LRR genes of *S. arcanum* LA2157, *S. pimpinellifolium* LA2093 and *S. lycopersicum* Heinz 1706 on chromosome 6 are shown in Figure 3a. CT119, REX and C8B were reported as PCR‐based markers linked to *Mi‐9* (Ammiraju *et al*., 2003). Chromosome 6 of LA2157 was 55.10 Mb in length (Figure 3b), the distance between CT119 and C8B occupied 12.96 Mb at the end of chromosome 6 in LA2157, and the NBS‐LRR genes were mainly distributed in the region of 706 kb (Figure 3b). There were seven NBS‐LRR genes (i.e., *Sarc_034231*, *Sarc_034228*, *Sarc_034201*, *Sarc_034200*, *Sarc_034198*, *Sarc_034196* and *Sarc_034194*) located between CT119 and C8B (Figure 3b, Table S7). Previous reports showed that the complete gene structure of *Mi‐1* included CC, NBS and LRR domains (Milligan *et al*., 1998). As a homologue of *Mi‐1*, seven candidate *Mi‐9* genes (i.e., *Sarc_034194*, S*arc_034196*, *Sarc_034198*, *Sarc_034200*, *Sarc_034201*, *Sarc_034228 and Sarc_034231*) all contained complete NBS‐LRR domains.

A comparative analysis of 706 kb genomic region of LA2157 was performed through comparison with published genomes of *S. lycopersicum* Heinz 1706 and *S. pimpinellifolium* LA2093. Only one NBS‐LRR gene (*Solyc06g008730.3*) was annotated in the corresponding region of the cultivated tomato Heinz 1706 genome, and no NBS‐LRR genes were annotated in the annotated version of the published LA2093 genome. However, when mapping the seven LA2157 NBS‐LRR genes to the genomes of Heinz 1706 and LA2093, highly homologous genome fragments of the seven NBS‐LRR genes can be detected in their genomes, indicating the number of NBS‐LRR genes in the genome region were underrepresented in Heinz 1706 and LA2093. Therefore, we carried out a manual annotation in the genome region, and five and six other NBS‐LRR genes were identified in Heinz 1706 and LA2093, respectively (Table S8). To explore the evolutionary relationship of NBS‐LRR, we performed microsynteny analysis of the coding genes in the 706 kb genomic fragments. As shown in Figure 3b, most genes in the region among three genomes are collinear genes, while a 284 kb genomic structure variation (inversion) can be found in LA2157 genome compared with Heinz 1706 and LA2093. It was noted that, except *Sarc_034200*, six NBS‐LRR genes of LA2157 form collinear gene pairs with their homologues in Heinz 1706 and LA2093, and display one‐to‐one orthologous relationship, suggesting *Sarc_034200* may be an additional NBS‐LRR gene copy in LA2157. Further, multiple sequences alignment and phylogeny evolution analysis showed that *Sarc_034200* had the highest similarity with the functional resistance gene *Mi‐1.2* (Figure 3c). Based on the above analysis, our results suggest that *Sarc_034200* may be the candidate *Mi‐9* gene.

The *Mi‐9* gene is a homologous gene of *Mi‐1.2* and has a heat‐stable resistance to RKNs compared to *Mi‐1.2* (Jablonska *et al*., 2007). Thus, gene structure analysis of the *Sarc_034200* gene and the sequence alignment with *Mi‐1.2* were conducted to determine whether there were critical domain differences between them. The length of the *Sarc_034200* gene was 3849 bp, and it encoded a protein of 1257 amino acids and contained an intron of 75 bp (Figure S3a). Through searching the InterProScan (http://www.ebi.ac.uk/interpro/), the amino acids structure showed that the conserved domains of Sarc_034200 contained typical NB‐ARC and LRR domains and a RX‐CC‐like structure (Figures 3d, S3a). Nucleic acid sequence alignment between *Mi‐1.2* and *Sarc_034200* showed that there were some SNPs (single nucleotide polymorphisms) in the CDS (coding sequence) sequence (Figure S3b). The RX‐CC_like and NB‐ARC domains of Mi‐1.2 and Sarc_034200 were the same length; whereas, the length of LRR regions differed, in which Sarc_034200 showed a slightly longer LRR domain (Figures 3d, S3c). The protein similarity between Mi‐1.2 and Sarc_034200 was 97.22%, and there were 35 amino acid substitutions between Mi‐1.2 and Sarc_034200 protein (Figure S3c).

### Identification of an *Mi‐9* candidate gene through monitoring root gene expression and TRV‐mediated VIGS

**Figure 4:**
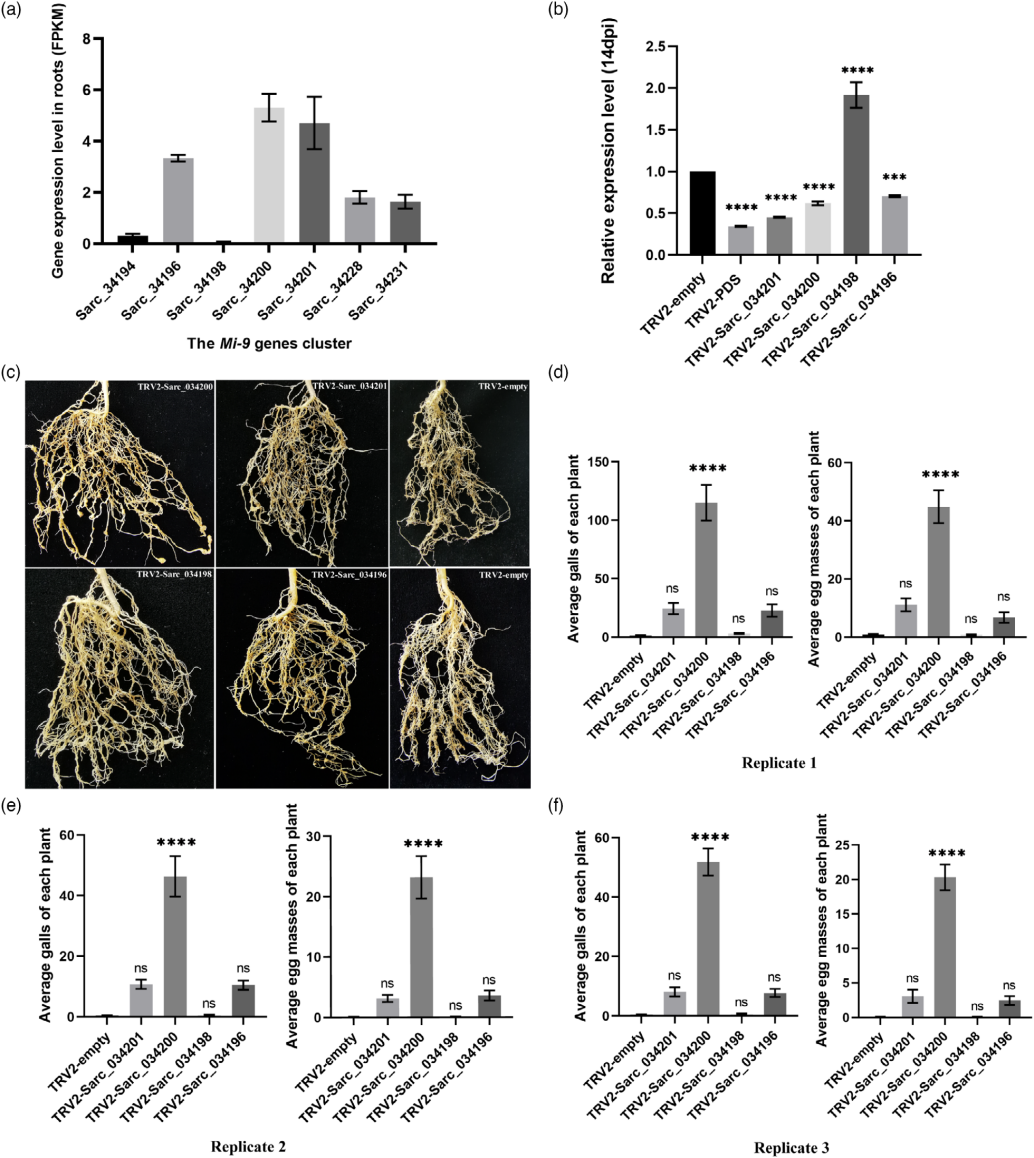
The gene expression level of *Mi‐9* genes cluster in the root tissue of LA2157 and the effect of tobacco rattle virus (TRV)‐mediated virus‐induced gene silencing (VIGS). (a) The transcriptional expression profiling of *Mi‐9* genes cluster in *M. incognita* inoculated LA2157 plants roots. The vertical axis indicates the Fragments Per Kilobase per Million (FPKM) of per gene. (b) The relative expression level of knockdown genes in LA2157 plants after VIGS treatment. qRT‐PCR was performed to detect the gene relative expression level at 14 days post infiltration. The *ubiquitin* (*UBI*) gene was used for normalization. The bar chart represents the mean values ± SD. The error bars represent SDs (***P < 0.001). Data were analysed by one‐way ANOVA followed by Dunnett's multiple comparisons test. (c)The root phenotypes of *Sarc_034196* gene, *Sarc_034198* gene, *Sarc_034200* gene and *Sarc_034201* gene silenced LA2157 plants at 40 days post inoculation (dpi), the plants infiltrated with TRV1 and TRV2‐empty were set as control. (d–f) Average galls and egg masses of three independent biological replicates of VIGS‐treated plants (>15 plants) at 40 days post‐inoculation. The bar chart represents the mean values ± SEM. The error bars represent SEMs (****P < 0.0001). Data were analysed by one‐way ANOVA followed by Dunnett's multiple comparisons test.

To identify the correct *Mi‐9* gene, transcriptional expression profiling of *Mi‐9* genes cluster in root tissue of LA2157 plants was conducted to narrow the screening range of candidate genes by excluding unexpressed genes. Fragments Per Kilobase per Million (FPKM) was used to represent the expression levels of seven candidate *Mi‐9* genes in LA2157 root tissues (Figure 4a). The expression level of *Sarc_34200* was the highest, followed by those of *Sarc34201* and *Sarc_34196*; whereas, the expression levels of *Sarc_34231* and *Sarc_34228* were low. *Sarc_34198* and *Sarc_34194* were barely expressed, they may be two unexpressed genes.

VIGS experiments were performed to preliminary screen and identify the function of candidate *Mi‐9* genes in nematodes susceptibility. Compared to the empty vector control (Figure S4a,c), an obvious photobleaching phenotype was observed in young leaves of plants injected with the TRV1 and TRV2‐*PDS* components after 3 weeks (Figure S4b), and severe photobleaching appeared in the plants 40 days after infiltration (Figure S4d), indicating the effectiveness of the gene silencing system in the VIGS experiment. The RT‐qPCR results, 2 weeks after infiltrating by *Agrobacterium tumefaciens*, showed that *PDS*, *Sarc_034201*, *Sarc_034200* and *Sarc_034196* genes were effectively silenced (approximately 30%–66%) with significant differences compared to the control (*P* ≤ 0.001; Figure 4b); whereas, *Sarc_034198* was not effectively silenced (Figure 4b), which was consistent with the assumption that it was an unexpressed gene.

VIGS‐treated plants were inoculated with *M. incognita* pre‐parasitic second‐stage juveniles (pre‐J2s) 2 weeks after infiltration. Root phenotypes were observed after 40‐d post‐infection as shown in Figure 4c. Statistical analysis of three independent biological replicates showed significant increases (*P* < 0.0001) in the number of galls (81–129‐times higher than the control) and egg masses (50–304‐times higher than the control) in *Sarc_034200* silenced plants (Figure 4d–f). Although the numbers of galls and egg masses also increased slightly in *Sarc_034201* and *Sarc_034196* silenced plants, there was no significant difference compared to the control. For *Sarc_034198*, which was predicted to be an unexpressed gene, silenced plants showed the same resistant phenotype as the control group. These results suggested that *Sarc_034200* is the candidate *Mi‐9* gene.

### Resistance of transgenic *S. pimpinellifolium* plants with the *Sarc_034200* gene to *M. incognita* under a moderate temperature

To further confirm that *Sarc_034200* was the *Mi‐9* gene, *S. pimpinellifolium* PI365967 (i.e. a tomato variety that is susceptible to *M. incognita*) was genetically transformed with the *Sarc_034200* gene. DNA‐level verification of T_0_ generation positive transgenic plants and mRNA‐level detection of 30 positive plants was conducted, which indicated that the *Sarc_034200* gene was successfully transformed into the *S. pimpinellifolium* plants and could be normally transcribed into mRNA (Figure S5). Moreover, RT‐qPCR results showed that the *Sarc_034200* gene was significantly expressed (approximately 3.8–102 times higher than the control) in T_0_ generation transgenic plants (Figure S6).

Three *Sarc_034200* transgenic *S. pimpinellifolium* T_1_ generation lines (line 1, line 2 and line 3) were used for resistance identification under a moderate temperature of 25 °C. After 40‐d post‐inoculation with *M. incognita*, phenotypic statistical analysis and PCR molecular detection were conducted (Figure S7; Table 1). As a result, 17 plants of line 1, 9 plants of line 2 and 14 plants of line 3 showed a high resistance to *M. incognita* (Table 1); conversely, 5 plants of line 2 and 2 plants of line 3 were susceptible to *M. incognita* (Table 1). There were also several plants for which PCR tests were negative but their phenotype was resistant; these may have been inoculated ineffectively. For non‐transgenic controls, 20 wild *S. pimpinellifolium* plants all showed high susceptibility, and 15 LA2157 plants were all resistant (Table 1).

**Table 1:** Phenotypic statistics of three T1 generation transgenic lines of *Sarc_034200*

Phenotypic statistics (number of plants corresponding to each phenotype)		Resistance to root‐knot nematodes at a high temperature of 30 °C		Resistance to root‐knot nematodes at a moderate temperature of 25 °C	
		R (resistance)	S (sensitive)	R (resistance)	S (sensitive)
Line 1	PCR (+)	18	0	17	0
	PCR (−)	0	0	0	0
Line 2	PCR (+)	11	0	9	0
	PCR (−)	4 (ineffective inoculations)	3	4 (ineffective inoculations)	5
Line 3	PCR (+)	21	0	14	0
	PCR (−)	1 (ineffective inoculations)	0	2 (ineffective inoculations)	2
Non‐transgenic negative control	PCR (+)	0	0	0	0
	PCR (−)	0	19	0	20
LA2157 positive control	PCR (+)	17	0	15	0
	PCR (−)	0	0	0	0

**Figure 5:**
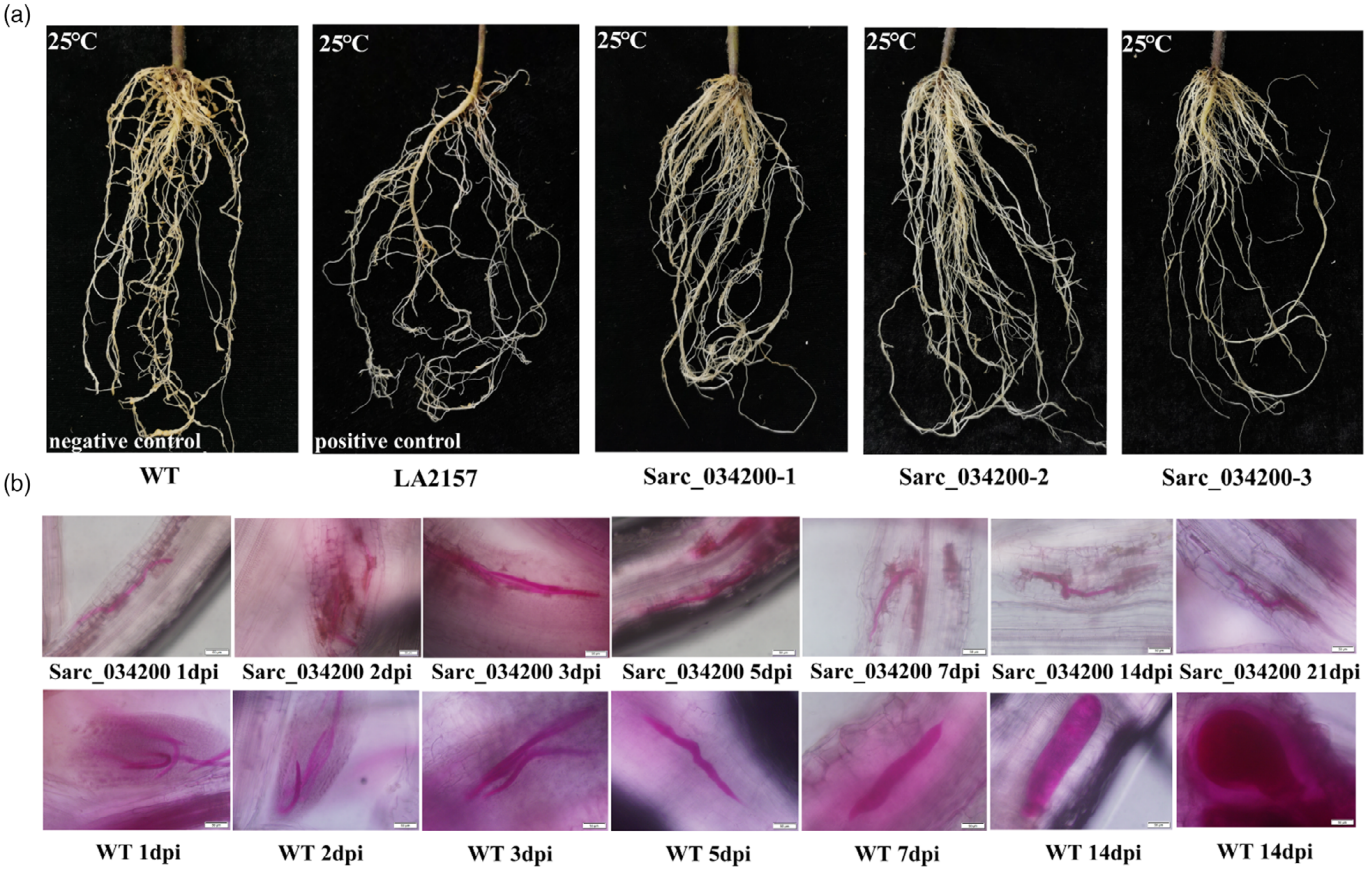
Phenotypes of *Sarc_034200* transgenic *S. pimpinellifolium* T1 generation plants after 40 days post inoculation (dpi) under 25 °C. (a) The root phenotypes of three *Sarc_034200* T1 generation transgenic lines after 40 dpi under 25 °C, non‐transgenic wild‐type *S. pimpinellifolium* plants were set as the negative control and LA2157 plants were set as the positive control. The positive plants of three T1 generation transgenic positive line showed high resistance to *M. incognita* as did the positive control plants, while the negative control plants showed high susceptibility. (b) Developmental status of *M. incognita* by staining with acid fuchsin within 3 weeks after infection of roots of *Sarc_034200* T1 generation transgenic plants under 25 °C. Wild‐type *S. pimpinellifolium* plants were set as the control. And the images were taken at 40× magnification. The nematodes were unable to grow and develop normally in *Sarc_034200* transgenic plants and a hypersensitive response was found near the infection site, while the nematodes could maintain normal growth and develop into adult females in the control plants.

Overall, approximately 75% of the three T_1_ generation transgenic plants were confirmed to carry *Sarc_034200* and all showed high resistance compared to non‐transgenic *S. pimpinellifolium* control plants, as did almost all of the LA2157 plants. The comparison of representative symptoms of roots is shown in Figure 5a. After inoculation with *M. incognita* for 3 weeks, the acid fuchsin stained roots of *Sarc_034200* T_1_ generation transgenic plants showed that nematodes were unable to develop normally in these plants and hypersensitivity response was found near the infection site; whereas, the nematodes could maintain normal growth and many developed into adult females in the wild *S. pimpinellifolium* plants (Figure 5b).

### Stable RKN‐resistance of transgenic *S. pimpinellifolium* plants with the *Sarc_034200* gene under 30 °C

To verify whether the *Sarc_034200* gene had a stable resistance under a high temperature, the three *Sarc_034200* T_1_ generation transgenic lines were used for further resistance identification under a high temperature of 30 °C. PCR molecular detection was conducted after 40‐d post‐inoculation with *M. incognita* (Figure S7), and phenotypic statistical analysis showed that 18 plants of line 1, 11 plants of line 2 and 21 plants of line 3 showed a high resistance (Table 1). There were also three plants of line 2 that appeared susceptible to *M. incognita* and several ineffective inoculations (Table 1).

**Figure 6:**
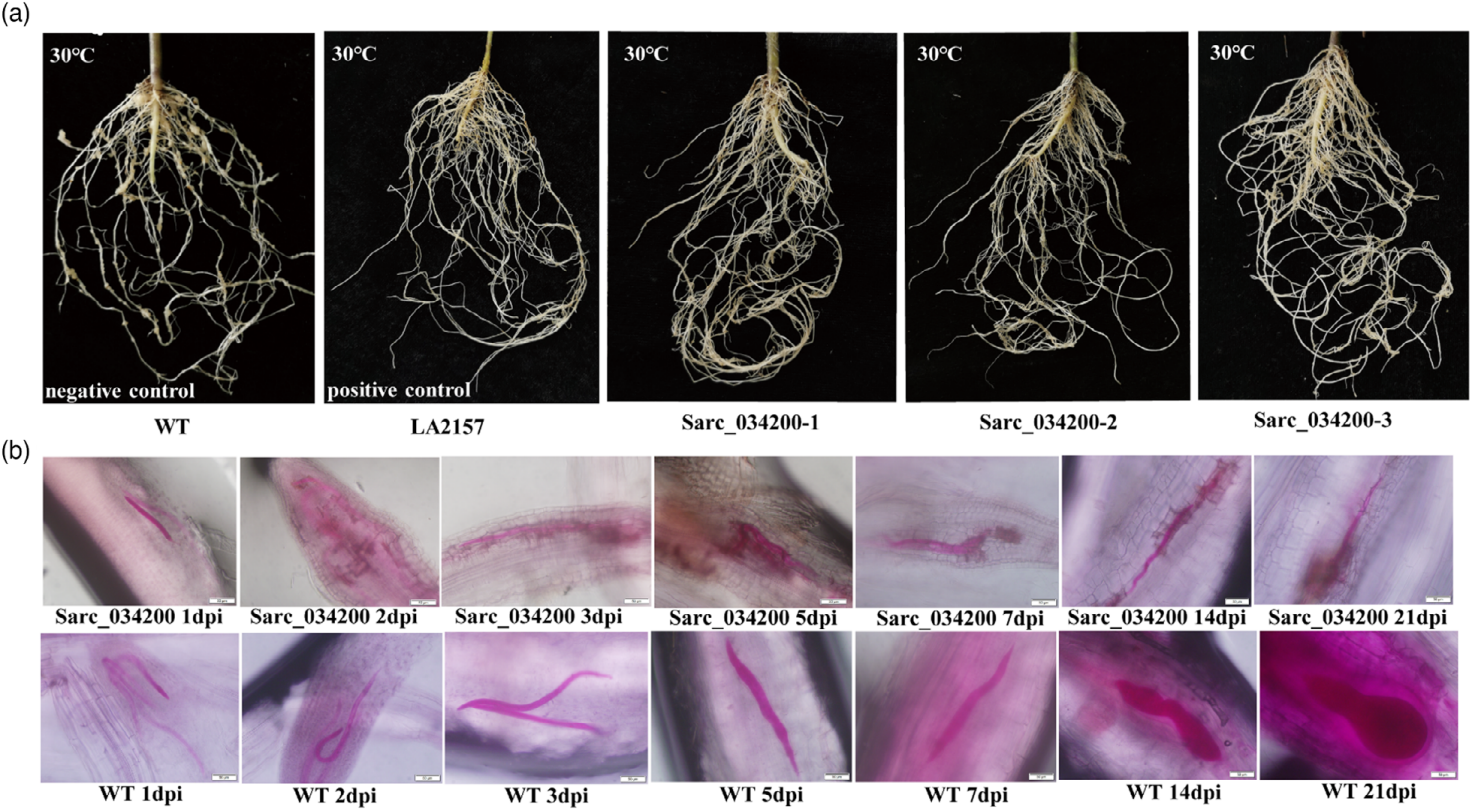
Phenotypes of *Sarc_034200* transgenic *S. pimpinellifolium* T1 generation plants after 40 days post inoculation (dpi) under 30 °C. (a) The root phenotypes of three *Sarc_034200* T1 generation transgenic lines after 40 dpi under 30 °C, non‐transgenic wild‐type *S. pimpinellifolium* plants were set as the negative control and LA2157 plants were set as the positive control. The positive plants of three T1 generation transgenic positive line showed high resistance to *M. incognita* as did the positive control plants, while the negative control plants showed high susceptibility. (b) Developmental status of *M. incognita* by staining with acid fuchsin within 3 weeks after infection of roots of *Sarc_034200* T1 generation transgenic plants under 30 °C. Wild‐type *S. pimpinellifolium* plants were set as the control, and the images were taken at 40x magnification. The nematodes were unable to grow and develop normally in *Sarc_034200* transgenic plants and a hypersensitive response was found near the infection site, while the nematodes could maintain normal growth and develop into adult females in the control plants.

Collectively, approximately 86% of the three T_1_ generation transgenic plants were confirmed to carry *Sarc_034200* and all showed high resistance, as did all the LA2157 plants; whereas, all wild *S*. *pimpinellifolium* plants showed high susceptibility. Representative symptoms of roots are shown in Figure 6a. Acid fuchsin‐stained roots after inoculation with *M. incognita* for 3 weeks showed that nematodes were also unable to develop normally in *Sarc_034200* transgenic plants, and hypersensitivity response was found near the infection site under 30 °C. However, the nematodes developed into adult females in the wild *S. pimpinellifolium* control group (Figure 6b). Overall, it was concluded that *Sarc_034200* is the *Mi‐9* gene and that it conferred stable resistance to *M. incognita* under a high temperature.

## Discussion

The discovery of plant disease resistance genes and research into the mechanisms have always been of far‐reaching significance for improving crop quality. Since *Mi‐1* was successfully cloned, it has been widely used in tomato production to counteract RKNs (Gilbert and Mcguire, 1956; Smith, 1944); however, resistance breaks down when the soil temperature exceeds 28 °C (Dropkin, 1969; Holtzmann, 1965). Therefore, cloning and application of genes with high‐temperature resistance for RKNs control is an urgent problem that needs to be solved in tomato production. *R* genes of different classes could be clustered in the same region of chromosome, as in previous studies, the conserved intron flanking sequence of *Mi‐1* were used for amplification of *Mi‐1* homologues in *Mi‐9* donor *S. arcanum* accession LA1257, and confirmed that *Mi‐9* is homologous to *Mi‐1* by VIGS assay (Jablonska *et al*., 2007). Through that, gene identification is made possible by transforming the *R* gene into susceptible genotypes, but the full length of *Mi‐9* was not obtained; however, it is difficult to transfer to cultivars due to the incompatibility of hybrids between distant tomato species (Jablonska *et al*., 2007; Veremis *et al*., 1999). In this study, we obtained chromosome‐level *S. arcanum* LA2157 genome assembly through ONT long reads, Illumina short reads and Hi‐C chromatin contact information. We obtained 10.9 Mb of contig N50, which is relatively good for Nanopore reads assembly in *Solanum* corps when compared with 21 tomato Nanopore sequencing assembly in NCBI (under PRJNA865981). The high‐quality *S. arcanum* genome, which had high contiguity and completeness, can be used to study the evolution of *Solanum* species, along with the identification of genes associated with important agronomic traits, including high‐yield and disease‐resistant genes (TomatoGenomeConsortium, 2012). We carried out comparative genome analysis among *S. arcanum*, *S. lycopersicum* and *S. pimpinellifolium*. Three genomes displayed highly collinear relationships, while some structure variations including inversions and translocations were detected. Structure variations are one of the causes of the appearance and disappearance of new genes (Wendel *et al*., 2016), which can partly explain the differences in agronomic traits among the three genomes. Furthermore, by combining the reported molecular markers of *Mi‐9*, the *Mi‐9* genes cluster (including seven genes) was obtained. Our method avoided the time‐consuming and labour‐intensive aspects of traditional gene cloning methods such as map‐based cloning and realized the rapid and accurate discovery of the resistance gene.

The phylogenetic analysis of *Mi‐9* genes cluster with *Mi‐1* revealed that the amino acid sequence of Sarc_034200 had the highest similarity with Mi‐1.2. Protein structures analysis showed that Sarc_034200 had RX‐CC_like, NB‐ARC and LRR domains, and belonged to a typical resistance gene of the NBS‐LRR type. Sequence alignment showed that there were many SNPs in their CDS sequences and 35 amino acid substitutions between their proteins and that they had a protein similarity of 97.22%. Through monitoring root gene expression and TRV‐mediated VIGS, *Sarc_034200* was then preliminarily identified as the *Mi‐9* candidate gene. A sequence of approximately 9 kb, including the complete DNA sequence of *Sarc_034200*, was then cloned and transformed into *S. pimpinellifolium* for further functional verification. Approximately 75% of the three T_1_ generation transgenic plants under 25 °C and approximately 86% of the three T_1_ generation transgenic plants under 30 °C showed high resistance, as did all of the LA2157 plants; whereas, all the wild *S*. *pimpinellifolium* plants showed a high susceptibility. Overall, we conclude that the *Sarc_034200* gene had stable resistance to RKNs under both 25 °C and 30 °C, and hence that it is the *Mi‐9* gene. To the best of our knowledge, this is the first time that the *Mi‐9* gene has been cloned (El‐Sappah *et al*., 2019; Jablonska *et al*., 2007). More importantly, *S. pimpinellifolium* is considered the wild ancestor of the cultivated tomato, and can be directly hybridized with cultivated tomato; therefore, the successful transformation of *Mi‐9* into *S. pimpinellifolium* would be of great significance for breeding RKN‐resistant tomato plants.

*Mi‐1* is a member of the plant resistance gene of the CC‐NBS‐LRR class, the RKN resistance of which shows a typical hypersensitive response of localized cell death around the nematode feeding site (Williamson and Kumar, 2006). In this study, the acid fuchsin‐staining experiments showed that *M. incognita* was unable to grow and develop normally in *Sarc_034200* transgenic plants and a hypersensitive response was found near the infection site, indicating that *Sarc_034200* conferred resistance to *M. incognita*. A previous study on the role analysis of extended N terminus for *Mi‐1.2* activation showed that Solanaceae domain 1 (SD1) could be assigned to a negative regulatory function and that the Solanaceae domain 2 (SD2) and coiled‐coil (CC) domain function as positive co‐regulators of *Mi‐1.2*‐mediated cell death (Lukasik‐Shreepaathy *et al*., 2012). As the most variable regions of closely related R genes, the LRR region of *Mi‐1.2* is related to the transmission of the resistance response and nematode recognition (Hwang and Williamson, 2003). We speculated that the LRR or other regions of *Mi‐9* may perform the same function as *Mi‐1*; however, further research is needed to confirm this and determine whether there are corresponding avirulence genes in the nematodes. In addition, it has been reported that the *Mi‐1.2* gene, but not the *Mi‐1.1* gene, exhibits anti‐nematode activity (Milligan *et al*., 1998). Previous studies have also shown that the difference in anti‐nematode activity between *Mi‐1.1* and *Mi‐1.2* activities was mainly caused by variation in the LRR region (Hwang *et al*., 2000). Further studies are needed to determine whether the difference in high‐temperature stability between the two genes is caused by the LRR domain. As an essential component in many *R* gene‐mediated resistance responses, salicylic acid (SA) is reported as an important part of signalling that leads to the *Mi‐1*‐mediated resistance and associated hypersensitive response to RKNs (Branch *et al*., 2004). JA could also induce RKN tolerance in tomatoes (Bali *et al*., 2017). *Mi‐1* is inactivated when the soil temperature is over 28 °C, and the resistant mechanism of *Mi‐1* is complex. Rem1 protein may be the target of attack when threatened by nematodes or other pests; ATP then binds to the Mi‐1 protein and is hydrolyzed, promoting the Mi‐1 protein to form signal bodies with HSP90‐1 and Sgt1 to active signal transduction (Bhattarai *et al*., 2007). Therefore, it was speculated that activation of downstream disease resistance pathways, such as the hormone signalling pathway, may also play a certain role in high‐temperature stable resistance in *Mi‐9*.

In conclusion, we cloned the full‐length DNA sequence of *Mi‐9* and verified its stable resistance to RKNs under a high temperature of 30 °C. Future studies are needed to study the high‐temperature resistance mechanism and further develop the genetic transformation of cultivated tomatoes. Our research lays a foundation for the creation of tomato germplasm resources that are resistant to RKNs under a high temperature.

## Experimental procedures

### Plant and nematode materials

*Solanum arcanum* (accession LA2157) leaves were collected and used for Illumina, Oxford Nanopore, Hi‐C sequencing and transcriptome sequencing. Root tissue was used for further RNA‐seq sequencing to explore the gene expression. *Solanum arcanum* LA2157 plants at the seedling stage were used to conduct a virus‐induced gene silencing (VIGS) assay for identification of the gene function. *Solanum pimpinellifolium* (PI365967) were used for genetic transformation of the candidate *Mi‐9* gene. All plant materials were grown in a greenhouse. *Meloidogyne incognita* were propagated on pepper (*Capsicum annuum*, Qiemen) in greenhouse from a single female egg mass isolated from Sijiqing farm (Beijing, China) (Shi *et al*., 2018). *Meloidogyne incognita* egg masses were collected and hatched in water. All nematode materials used in this study were *M. incognita* pre‐parasitic second‐stage juveniles (pre‐J2s) and were inoculated in three holes near the root zone as specified (Lizardo *et al*., 2022).

### Genome sequencing, long‐range chromosome assembly and gene annotation

High‐quality DNA was prepared using the CTAB method (Kuo *et al*., 2022), followed by purification using a QIAGEN Genomic kit (Cat#13443; Qiagen, Dusseldorf，Germany). The purified genomic DNA was then used to construct the 1D library using SQK‐LSK109 (Oxford Nanopore Technologies, Oxford Science Part, UK) according to the manufacturer's instructions, and ONT sequencer Nanopore PromethION was used for DNA single‐molecule sequencing to obtain the original sequencing data. The Nanopore original sequence was assembled with NextDenovo (https://github.com/Nextomics/NextDenovo) v2.0.0 after quality control and filtering of low‐quality sequences. The contigs obtained by assembly were polished by NextPolish (Hu *et al*., 2020) error correction and then by two rounds of Racon (Vaser *et al*., 2017) to obtain the preliminary assembly results. Purge Haplotigs (Roach *et al*., 2018) was used to diploidized the genome to obtain haploid sequences. Clean reads of Hi‐C sequences were obtained after quality control, which were pre‐processed and mapped to contigs by juicer (Durand *et al*., 2016a). Genome error correction and assembly were then performed by a 3D‐DNA process (Dudchenko *et al*., 2017), and manual inspection and adjustment were finally performed by a juicer box (Durand *et al*., 2016b) to obtain the final genome assembly version.

A strategy combining ab initio gene prediction, homology‐based gene prediction and RNA‐seq was used for gene annotation. The repetitive sequences were annotated by combining ab initio and homology‐based methods. First, an ab initio repeat library was predicted for each genome with RepeatModeler2 (Flynn *et al*., 2020). Second, this library was combined with Repbase (Jurka *et al*., 2005) (http://www.girinst.org/repbase) to identify all homologous repeats throughout the genome by RepeatMasker (Tarailo‐Graovac and Chen, 2009) (http://www.repeatmasker.org/) with BLASTX (Tarailo‐Graovac and Chen, 2009) as the search engine. The RNA‐seq reads were assembled into contigs using Trinity (Grabherr *et al*., 2013) with default parameters and further predicted gene structures using PASA (Haas *et al*., 2003). We trained Augustus (Stanke *et al*., 2006) and SNAP (Korf, 2004) using the high confident gene models from the results of the PASA assembly, and GeneMark‐ES (Lomsadze *et al*., 2014) were self‐trained on the repeat‐masked genome sequences. Homologous protein sequences from *Arabidopsis thaliana*, *S. lycopersicum*, *Solanum. pennellii*, *Solanum tuberosum*, *C. annuum* and *Solanum melongena* were downloaded from Phytozome (https://phytozome‐next.jgi.doe.gov/), and were mapped to each assembly with TBLASTN (Gertz *et al*., 2006) with an e‐value threshold of 1e‐5. Genewise (Birney *et al*., 2004) (parameter: ‐gff ‐quiet ‐silent ‐sum) was used to refine the alignment. All results were integrated into consensus gene models using EvidenceModeler (Haas *et al*., 2008). The whole genome was aligned with AnchorWave (Song *et al*., 2022), and the collinearity and structural variation regions were then identified by SyRI (Synteny and Rearrangement Identifier) (Goel *et al*., 2019) and displayed by IGV (Thorvaldsdóttir *et al*., 2013) and PlotsR (Goel *et al*., 2022).

To identify the NBS‐LRR, we downloaded the annotation data of tomatoes from Sol Genomics Network (https://solgenomics.net/). The sequences were searched using hmmsearch of HMMER (v3.3.2) with the raw Hidden Markov Model (HMM) NB‐ARC family (PF00931) (Potter *et al*., 2018). Proteins with an ‐E 1e‐10 were selected to construct a NBS HMM profile using hmmbuild and then searched NBS‐LRR genes in LA2157 protein sequences with an e‐value threshold of 1e‐10. The TIR, NBS, CC and LRR domains of the identified NBS–LRR proteins were confirmed using Pfam (http://pfam.sanger.ac.uk/) and SMART (http://smart.embl‐heidelberg.de/). To avoid underestimating number of NBS‐LRR in the genome, NLR‐Annotator (v2.0) (Zhang, 2020) was used for genome‐wide identification of NLR gene loci. The loci that contained NLR‐like motifs but were not annotated as genes were annotated manually. The classification of NBS‐LRR was carried out using PRGdb (http://prgdb.org/prgdb/) (Osuna‐Cruz *et al*., 2018).

### Mapping and distribution of *Mi‐9* genes cluster on chromosome 6 of LA2157 and comparative genomic analysis with *S. lycopersicum* Heinz1706 and *S. pimpinellifolium* LA2093

The preliminary localization of *Mi‐9* gene clusters on chromosome 6 of *S. arcanum* LA2157 genome was located based on molecular markers of *Mi‐9* gene, and Apollo (Lewis *et al*., 2002) was used to manually check and edit genes in the *Mi‐9* gene located region. To locate the homologous genes of *Mi‐9* in different genomes as comprehensively as possible, FGF (Zheng *et al*., 2007) was used to manually predict and identify the candidate *Mi‐9* gene family. The amino acid sequence of the candidate *Mi‐9* gene was used as the query sequence and TBLASTN was conducted to search the genome sequence for preliminary localization. GeneWise was then used to predict the gene structure. Sequences alignment and phylogeny analysis of *Mi‐9* genes cluster, *Mi‐1* and the genes in the same chromosome segment in *S. lycopersicum* Heinz 1706 and *S. pimpinellifolium* LA2093 was conducted by MEGA7 software using neighbour‐joining method.

### Expression analysis of the *Mi‐9* genes cluster in transcriptome of LA2157 root

The root tissue of LA2157 plants inoculated with *M. incognita* was collected after 7 days, and three biological replicates were set up. Samples were then transported to Nextomics Bioscience Co., Ltd. (Wuhan, China) for the transcriptome sequencing after freezing using liquid nitrogen. RNA was extracted through CTAB‐LiCl, and the ordinary transcriptome library of mRNA was constructed after passing quality inspection following the previously published protocol (Chang *et al*., 1993; Ling *et al*., 2017). 150PE sequencing was then performed on a MGISEQ‐T7 sequencer (MGI, Shenzhen, China), and the data volume of each sample was ≥6 Gb of raw data. Tophat (Trapnell *et al*., 2012) software was used to locate the sequenced reads onto the sequenced genome with the parameter ‐r 30‐p 20. Cufflinks (Trapnell *et al*., 2012) were then used to link reads with annotated genes, and the expression levels of each gene were obtained via HTSeq‐count.

### Identification of the *Mi‐9* candidate gene by VIGS

A 300–400 bp fragments of four *Mi‐9* candidate genes (i.e., the accessions named *Sarc_034201*, *Sarc_034200*, *Sarc_034198* and *Sarc_034196*) were cloned by PCR using a 2xPhanta Flash Master Mix (Vazyme, Nanjing, China) and the template cDNA was obtained though using a Thermo Scientific ReverAid First Strand cDNA Synthesis Kit K1622 (Waltham, MA, USA). The primer pairs were designed using the Primer3.0 v.0.4.0 (https://bioinfo.ut.ee/primer3‐0.4.0/) website and are shown in Table S9. The amplified fragments were then constructed into the tobacco rattle virus RNA2 (TRV2) vector digested by endonuclease *EcoR*I (NEB, Ipswich, MA, USA) and *BamH*I (NEB). A mixture of *A. tumefaciens* containing TRV1 and TRV2 with corresponding fragments was used to infiltrate into leaves of LA2157 tomato seedlings at the 4–6 leaves stage. The *phytoene desaturase* (*PDS*) gene of tomato was used as a positive control to show a successful gene silencing (Liu *et al*., 2002) and *A. tumefaciens* carrying TRV1 and empty TRV2 was used as a negative control.

Root tissues of treated plants were collected and RNA was extracted at 14‐days post infiltration. Reverse transcription‐quantitative real‐time PCR (RT‐qPCR) was then conducted to detect the efficacy of gene silencing using HiScript® III RT SuperMix for qPCR (+gDNA wiper) (Vazyme) and Taq Pro Universal SYBR qPCR Master Mix (Vazyme) according to the manufacturer's instructions. qPCR was performed on a Bio‐Rad CFX96 (Hercules, CA, USA) real‐time PCR system with the following amplification program: 95 °C for 10 min and 40 cycles of 95 °C for 10 s and 60 °C for 30 s. The primer pairs were designed using the Primer3.0 website and are shown in Table S10, and tomato *ubiquitin* (*UBI*) was used as a reference for the normalization of gene expression. Data were calculated using the 2^−ΔΔCT^ method (Livak and Schmittgen, 2001). Three independent biological replicates experiments were conducted, and each reaction was set with four technical replicates (Zhao *et al*., 2021). The VIGS‐treated plants were then inoculated with 800 *M. incognita* pre‐J2s after 2 weeks post infiltration for each plant. Root phenotypes were then observed, and galls and egg masses were counted at 40‐days post‐inoculation. Three independent biological replicate experiments were conducted and > 15 plants were checked in each treatment.

### Cloning and genetic transformation of *Sarc_034200*

The *Sarc_034200* gene, which was identified as the candidate *Mi‐9* gene, was cloned by BAC screening and long PCR amplification using the primer pairs clone‐*Sarc_034200*‐F/R shown in Table S11. An approximately 9 kb‐length amplicon including the complete DNA sequence, approximately 4 kb upstream sequence of the potential promoter region and approximately 1 kb downstream sequence of the termination site, was attached to the pBINPLUS vector digested by *Sal*I‐HF (NEB) and *Pac*I‐HF (NEB). The sequenced recombinant plasmid was then transformed into *A. tumefaciens* strain *AGL1*. Genetic transformation of the *S*. *pimpinellifolium* PI365967 (i.e., a tomato variety susceptible to RKNs) was conducted using the *A. tumefaciens*‐mediated method (Wang *et al*., 2021).

### Screening and resistance identification of the *Sarc_034200* transgenic plants

DNA of *Sarc_034200* T_0_ generation transgenic plants were extracted using a plant genome DNA extraction kit (TianGen, Beijing, China). The *Sarc_034200* gene was identified by PCR molecular detection using the gene‐specific primer pairs Trans‐DNA Sense/Antisense shown in Table S11. RNA of the positive T_0_ generation transgenic seedlings was extracted using a RNAprep Pure plant total RNA extraction kit (TianGen, Beijing, China), and cDNA was synthesized using HiScript^®^ III RT SuperMix for qPCR (+gDNA wiper) (Vazyme). The transcription of *Mi‐9* gene was verified using the primer pairs shown in Table S11, and RT‐qPCR was used to detect the gene relative expression level using the *q‐Sarc_034200* primer pairs and the *UBI* primer pairs shown in Table S10. Three of positive T_1_ transgenic lines were inoculated with *M. incognita* for the subsequent resistance assay. LA2157 plants were used as positive control and wild‐type *S. pimpinellifolium* plants were used as a negative control. To determine whether the candidate *Mi‐9* gene showed resistance to *M. incognita* at a high temperature, two temperature treatments were designed: 40 plants at the 4–6 leaves stage were separated for the resistance assay at a moderate temperature of 25 °C and a high temperature of 30 °C, and each plant was inoculated with 800 *M. incognita* pre‐J2s. Phenotypic and statistical analysis were investigated after 40‐days post‐inoculation. Nematode development in root tissues of T_1_ generation transgenic plants and wild *S. pimpinellifolium* plants (control) was visualized by acid fuchsin staining at different time points (24 h, 48 h, 3, 5, 7, 14 and 21 days), and photographs were taken using an inverted microscope (OLYMPUS IX53, ×40 objective, Japan).

### Data analysis

Statistical analysis of RT‐qPCR, numbers of galls and egg masses were conducted via one‐way ANOVA followed by Dunnett's multiple comparisons tests using GraphPad Prism8.0 (Zhao *et al*., 2021).

## Author contributions

B. X., Z. M., J. Z. and L. J., designed the research; Y. W. and J. L. analysed the bioinformatics data; L. J., Y. Y., Y. L. Y. J. and J. Z., performed the experiments and analysed data. Y. Y. constructed the BAC library. L. J., J. L. J. Z. and Y. W. wrote the manuscript. All authors commented on the manuscript before submission.

## Conflict of interest

The authors declare that there are no conflicts of interest related to this manuscript.

## Supporting information


**Figure S1** Genome size estimate using GenomeScope. The genome size, heterozygosity and repeat content were estimated using GenomeScope. About 50 × NGS (Illumina) reads were used to count the k‐mer and export the k‐mer count histogram using jellyfish. The genome size of LA2157 was estimated to be 672.9 Mb in length using GenomeScope method.
**Figure S2** Whole genome alignment among *S. arcanum* LA2157, *S. lycopersicum* Heinz 1706 and *S. pimpinellifolium* LA2093. Grey curve connects the syntenic regions between three genomes. Numbers represent the chromosome numbers.
**Figure S3** Gene structure of *Sarc_034200* and sequence alignment of *Sarc_034200* and *Mi‐1.2*. (a) Gene structure and conserved domains of *Sarc_034200*. (b) CDS Sequence alignment of *Sarc_034200* and *Mi‐1.2*. (c) Amino acid sequence alignment of *Sarc_034200* and *Mi‐1.2*, the arrows represent the conserved domains.
**Figure S4** Phenotypes of young leaves of LA2157 plants infiltrated by TRV1 component and TRV2‐*PDS* component. Phenotypes of plants infiltrated by TRV1 and TRV2‐*PDS*, and TRV1 and TRV2‐empty infiltrated plants were set as control. (a) and (c) control plants infiltrated by TRV1 and TRV2‐empty. (b) plants of 3 weeks after infiltrated by TRV1 and TRV2‐*PDS*. (d) plants of 40 days after infiltrated by TRV1 and TRV2‐*PDS*.
**Figure S5** DNA and mRNA level detection of *Sarc_034200* T_0_ generation transgenic plants. (a, b) DNA level detection of *Sarc_034200* T_0_ generation transgenic plants. +, positive control (plasmid); −, negative control (WT plant). (c) mRNA level detection of 30 *Sarc_034200* T_0_ generation transgenic plants. +, positive control (plasmid); −, negative control (WT plant).
**Figure S6** Gene relative expression level of *Sarc_034200* in 30 T_0_ generation transgenic seedlings. RT‐qPCR was conducted to detect the gene‐relative expression level of *Sarc_034200* in 30 T_0_‐generation transgenic plants. The *ubiquitin* (*UBI*) gene was used for normalization. The bar chart represents the mean values ± SD. The error bars represent SDs (***P < 0.001). Data were analysed by one‐way ANOVA followed by Dunnett's multiple comparisons test.
**Figure S7** DNA level detection of three *Sarc_034200* T_1_ generation transgenic lines. (a, c, e) Detection of *Sarc_034200* with specific primers in three *Sarc_034200* T_1_ generation transgenic lines under the temperature of 25 °C. (b, d, f) Detection of *Sarc_034200* with specific primers in three *Sarc_034200* T_1_ generation transgenic lines under the temperature of 30°C. (g, h) A repeat detection of the plants with no bands or weak bands. +, positive control (plasmid); −, negative control (WT plant).

**Table S1** Nanopore raw data and final assembled data of LA2157 genome.
**Table S2** Statistics of the LA2157 genome assembly and comparison with *S. lycopersicum* Heinz 1706 and *S. pimpinellifolium* LA2093.
**Table S3** The gene function annotation of LA1257.
**Table S4** Collinearity analysis between LA2157 and two susceptible species (Heinz 1706 and LA2093).
**Table S5** The genetic locus of NBS‐LRR genes in LA2157.
**Table S6** NBS‐LRR genes identified from the genome of LA2157, *S. pimpinellifolium* LA2093 and *S. lycopersicum* (*Sly*).
**Table S7** The gene function annotation of candidate *Sarc_034200* region.
**Table S8** NBS‐LRR genes identified from706 kb region of LA2157, *S. pimpinellifolium* LA2093 and *S. lycopersicum* Heinz 1706.
**Table S9** Primes for silenced fragments of *Sarc_034200* candidate genes.
**Table S10** qPCR primers for detection of the efficiency of gene silencing.
**Table S11** Primers for detection of *Sarc_034200* T_0_ generation transgenic plants.

## Data Availability

All the sequencing raw data of *Solanum arcanum* LA2157 were deposited in NCBI SRA database under submission access number SUB9388536 and with the SRA accession from SRR23010874 to SRR23010878. The assembled whole genome sequences of *Solanum arcanum* LA2157 were deposited in NCBI with accession number JAPXVU000000000.

## References

[pbi14055-bib-0001] Ammiraju, J. , Veremis, J. , Huang, X. , Roberts, P. and Kaloshian, I. (2003) The heat‐stable root‐knot nematode resistance gene *Mi‐9* from *Lycopersicon peruvianum* is localized on the short arm of chromosome 6. Theor. Appl. Genet. 106, 478–484. 12589548 10.1007/s00122-002-1106-y

[pbi14055-bib-0002] Arora, S. , Steuernagel, B. , Gaurav, K. , Chandramohan, S. , Long, Y. , Matny, O. , Johnson, R. *et al*. (2019) Resistance gene cloning from a wild crop relative by sequence capture and association genetics. Nat. Biotechnol. 37, 139–143. 30718880 10.1038/s41587-018-0007-9

[pbi14055-bib-0003] Bai, Y. and Lindhout, P. (2007) Domestication and breeding of tomatoes: what have we gained and what can we gain in the future? Ann. Bot.‐London. 100, 1085–1094. 10.1093/aob/mcm150 PMC2759208 17717024

[pbi14055-bib-0004] Bailey, D.M. (1941) The seedling test method for root‐knot‐nematode resistance. Proc. Am. Soc. Horticultural Sci. 38, 573–575.

[pbi14055-bib-0005] Bali, S. , Kaur, P. , Sharma, A. , Ohri, P. , Bhardwaj, R. , Alyemeni, M.N. , Wijaya, L. *et al*. (2017) Jasmonic acid‐induced tolerance to root‐knot nematodes in tomato plants through altered photosynthetic and antioxidative defense mechanisms. Protoplasma 255, 471–484. 28905119 10.1007/s00709-017-1160-6

[pbi14055-bib-0006] Bhattarai, K.K. , Li, Q. , Liu, Y. , Dinesh‐Kumar, S.P. and Kaloshian, I. (2007) The *Mi‐1*‐mediated pest resistance requires *Hsp90* and *Sgt1* . Plant Physiol. 144, 312–323. 17351050 10.1104/pp.107.097246 PMC1913790

[pbi14055-bib-0007] Birney, E. , Clamp, M. and Durbin, R. (2004) GeneWise and genomewise. Genome Res. 14, 988–995. 15123596 10.1101/gr.1865504 PMC479130

[pbi14055-bib-0008] Bolger, A. , Scossa, F. , Bolger, M.E. , Lanz, C. , Maumus, F. , Tohge, T. , Quesneville, H. *et al*. (2014) The genome of the stress‐tolerant wild tomato species *Solanum pennellii* . Nat. Genet. 46, 1034–1038. 25064008 10.1038/ng.3046 PMC7036041

[pbi14055-bib-0009] Branch, C. , Hwang, C.F. , Navarre, D.A. and Williamson, V.M. (2004) Salicylic acid is part of the *Mi‐1*‐mediated defense response to root‐knot nematode in tomato. Mol. Plant‐Microbe Interact. 17, 351–356. 15077667 10.1094/MPMI.2004.17.4.351

[pbi14055-bib-0010] Castagnone‐Sereno, P. , Danchin, E.G. , Perfus‐Barbeoch, L. and Abad, P. (2013) Diversity and evolution of root‐knot nematodes, genus *Meloidogyne*: new insights from the genomic era. Annu. Rev. Phytopathol. 51, 203–220. 23682915 10.1146/annurev-phyto-082712-102300

[pbi14055-bib-0011] Chang, S.T.A.U. , Puryear, J. and Cairney, J. (1993) A simple and efficient method for isolating RNA from pine trees. Plant Mol. Biol. Report. 11, 113–116.

[pbi14055-bib-0012] Devran, Z. and Elekçioğlu, I.H. (2004) The screening of F_2 plants for the root‐knot nematode resistance gene *Mi*, by PCR in tomato. Turk. J. Agric. For. 28, 253–257.

[pbi14055-bib-0013] Devran, Z. , Sogut, M.A. and Mutlu, N. (2010) Response of tomato rootstocks with the *Mi* resistance gene to *Meloidogyne incognita* race 2 at different soil temperatures. Phytopathol. Mediterr. 49, 11–17.

[pbi14055-bib-0014] Dropkin, V.H. (1969) Necrotic reaction of tomatoes and other hosts resistant to *Meloidogyne*: reversal by temperature. Phytopathol. Z. 59, 1632–1637.

[pbi14055-bib-0015] Dudchenko, O. , Batra, S.S. , Omer, A.D. , Nyquist, S.K. , Hoeger, M. , Durand, N.C. , Shamim, M.S. *et al*. (2017) De novo assembly of the *Aedes aegypti* genome using Hi‐C yields chromosome‐length scaffolds. Science 356, 92–95. 28336562 10.1126/science.aal3327 PMC5635820

[pbi14055-bib-0016] Durand, N.C. , Robinson, J.T. , Shamim, M.S. , Machol, I. , Mesirov, J.P. , Lander, E.S. and Aiden, E.L. (2016) Juicebox provides a visualization system for Hi‐C contact maps with unlimited zoom. Cell Syst. 3, 99–101. 27467250 10.1016/j.cels.2015.07.012 PMC5596920

[pbi14055-bib-0017] Durand, N.C. , Shamim, M.S. , Machol, I. , Rao, S.S.P. , Huntley, M.H. , Lander, E.S. and Aiden, E.L. (2016) Juicer provides a one‐click system for analyzing loop‐resolution Hi‐C Experiments. Cell Syst. 3, 95–98. 27467249 10.1016/j.cels.2016.07.002 PMC5846465

[pbi14055-bib-0018] El‐Sappah, A.H. , El‐awady, H. , Yan, S. , Qi, S. , Liu, J. , Cheng, G. and Liang, Y. (2019) Tomato natural resistance genes in controlling the root‐knot nematode. Genes‐Basel. 10, 925. 31739481 10.3390/genes10110925 PMC6896013

[pbi14055-bib-0019] Flynn, J.M. , Hubley, R. , Goubert, C. , Rosen, J. , Clark, A.G. , Feschotte, C. and Smit, A.F. (2020) RepeatModeler2 for automated genomic discovery of transposable element families. Proc. Natl. Acad. Sci. USA 117, 9451–9457. 32300014 10.1073/pnas.1921046117 PMC7196820

[pbi14055-bib-0020] Gertz, E.M. , Yu, Y.K. , Agarwala, R. , Schaffer, A.A. and Altschul, S.F. (2006) Composition‐based statistics and translated nucleotide searches: improving the TBLASTN module of BLAST. BMC Biol. 4, 41. 17156431 10.1186/1741-7007-4-41 PMC1779365

[pbi14055-bib-0021] Gilbert, J.C. and Mcguire, D.C. (1956) Inheritance of resistance to severe root‐knot from *Meloidogyne incognita* in commercial type tomatoes. Proc. Am. Soc. Hort. Sci. 68, 437–442.

[pbi14055-bib-0022] Goel, M. , Sun, H. , Jiao, W. and Schneeberger, K. (2019) SyRI: finding genomic rearrangements and local sequence differences from whole‐genome assemblies. Genome Biol. 20, 277. 31842948 10.1186/s13059-019-1911-0 PMC6913012

[pbi14055-bib-0023] Goel, M. , Schneeberger, K. and Robinson, P. (2022) Plotsr: visualizing structural similarities and rearrangements between multiple genomes. Bioinformatics 38, 2922–2926. 35561173 10.1093/bioinformatics/btac196 PMC9113368

[pbi14055-bib-0024] Grabherr, M.G. , Haas, B.J. , Yassour, M. , Levin, J.Z. , Thompson, D.A. , Amit, I. , Adiconis, X. *et al*. (2013) Trinity: reconstructing a full‐length transcriptome without a genome from RNA‐Seq data. Nat. Biotechnol. 29, 644–652. 10.1038/nbt.1883 PMC3571712 21572440

[pbi14055-bib-0025] Grech‐Baran, M. , Witek, K. , Szajko, K. , Witek, A.I. , Morgiewicz, K. , Wasilewicz‐Flis, I. , Jakuczun, H. *et al*. (2020) Extreme resistance to *Potato virus Y* in potato carrying the *Ry* _ *sto* _ gene is mediated by a TIR‐NLR immune receptor. Plant Biotechnol. J. 18, 655–667. 31397954 10.1111/pbi.13230 PMC7004898

[pbi14055-bib-0026] Haas, B.J. , Delcher, A.L. , Mount, S.M. , Wortman, J.R. , Smith, R.K., Jr. , Hannick, L.I. , Maiti, R. *et al*. (2003) Improving the Arabidopsis genome annotation using maximal transcript alignment assemblies. Nucleic Acids Res. 31, 5654–5666. 14500829 10.1093/nar/gkg770 PMC206470

[pbi14055-bib-0027] Haas, B.J. , Salzberg, S.L. , Zhu, W. , Pertea, M. , Allen, J.E. , Orvis, J. , White, O. *et al*. (2008) Automated eukaryotic gene structure annotation using EVidenceModeler and the Program to Assemble Spliced Alignments. Genome Biol. 9, R7. 18190707 10.1186/gb-2008-9-1-r7 PMC2395244

[pbi14055-bib-0028] Holtzmann, O.V. (1965) Effect of soil temperature on resistance of tomato to root‐knot nematode (*Meloidogyne incognita*). Phytopathology 55, 990–992.

[pbi14055-bib-0029] Hosmani, P.S. , Flores‐Gonzalez, M. , van de Maumus, F. , Bakker, L.V. , Schijlen, E. , Haarst, J.V. , Cordewener, J. *et al*. (2019) An improved de novo assembly and annotation of the tomato reference genome using single‐molecule sequencing, hi‐c proximity ligation and optical maps. Cold Spring Harbor: Cold Spring Harbor Laboratory Press.

[pbi14055-bib-0030] Hu, J. , Fan, J. , Sun, Z. , Liu, S. and Berger, B. (2020) NextPolish: a fast and efficient genome polishing tool for long‐read assembly. Bioinformatics 36, 2253–2255. 31778144 10.1093/bioinformatics/btz891

[pbi14055-bib-0031] Hwang, C.F. and Williamson, V.M. (2003) Leucine‐rich repeat‐mediated intramolecular interactions in nematode recognition and cell death signaling by the tomato resistance protein Mi. Plant J. 34, 585–593. 12787241 10.1046/j.1365-313x.2003.01749.x

[pbi14055-bib-0032] Hwang, C.F. , Bhakta, A.V. , Truesdell, G.M. , Pudlo, W.M. and Williamson, V.M. (2000) Evidence for a role of the N terminus and leucine‐rich repeat region of the *Mi* gene product in regulation of localized cell death. Plant Cell 12, 1319–1329. 10948252 10.1105/tpc.12.8.1319 PMC149105

[pbi14055-bib-0033] Jablonska, B. , Ammiraju, J.S. , Bhattarai, K.K. , Mantelin, S. , Martinez, D.I.O. , Roberts, P.A. and Kaloshian, I. (2007) The *Mi‐9* gene from *Solanum arcanum* conferring heat‐stable resistance to root‐knot nematodes is a homolog of *Mi‐1* . Plant Physiol. 143, 1044–1054. 17172289 10.1104/pp.106.089615 PMC1803715

[pbi14055-bib-0034] Jones, J.T. , Haegeman, A. , Danchin, E.G. , Gaur, H.S. , Helder, J. , Jones, M.G. , Kikuchi, T. *et al*. (2013) Top 10 plant‐parasitic nematodes in molecular plant pathology. Mol. Plant Pathol. 14, 946–961. 23809086 10.1111/mpp.12057 PMC6638764

[pbi14055-bib-0035] Jurka, J. , Kapitonov, V.V. , Pavlicek, A. , Klonowski, P. , Kohany, O. and Walichiewicz, J. (2005) Repbase Update, a database of eukaryotic repetitive elements. Cytogenet. Genome Res. 110, 462–467. 16093699 10.1159/000084979

[pbi14055-bib-0036] Korf, I. (2004) Gene finding in novel genomes. BMC Bioinformatics. 5, 59. 15144565 10.1186/1471-2105-5-59 PMC421630

[pbi14055-bib-0037] Kuo, P. , Henderson, I.R. and Lambing, C. (2022) CTAB DNA extraction and genotyping‐by‐sequencing to map meiotic crossovers in plants. Methods Mol. Biol. 2484, 43–53. 35461443 10.1007/978-1-0716-2253-7_4

[pbi14055-bib-0038] Lewis, S.E. , Searle, S.M.J. , Harris, N. , Gibson, M. , Lyer, V. , Richter, J. , Wiel, C. *et al*. (2002) Apollo: a sequence annotation editor. Genome Biol. 3, H82. 10.1186/gb-2002-3-12-research0082 PMC151184 12537571

[pbi14055-bib-0039] Li, Q. , Li, H. , Huang, W. , Xu, Y. , Zhou, Q. , Wang, S. , Ruan, J. *et al*. (2019) A chromosome‐scale genome assembly of cucumber (*Cucumis sativus* L.). Gigascience 8, 1–10. 10.1093/gigascience/giz072 PMC6582320 31216035

[pbi14055-bib-0040] Ling, J. , Mao, Z. , Zhai, M. , Zeng, F. , Yang, Y. and Xie, B. (2017) Transcriptome profiling of *Cucumis metuliferus* infected by *Meloidogyne incognita* provides new insights into putative defense regulatory network in Cucurbitaceae. Sci. Rep. 7, 3515–3544. 28615634 10.1038/s41598-017-03563-6 PMC5471208

[pbi14055-bib-0041] Ling, J. , Xie, X. , Gu, X. , Zhao, J. , Ping, X. , Li, Y. , Yang, Y. *et al*. (2021) High‐quality chromosome‐level genomes of *Cucumis metuliferus* and *Cucumis melo* provide insight into *Cucumis* genome evolution. Plant J. 107, 136–148. 33866620 10.1111/tpj.15279

[pbi14055-bib-0042] Liu, Y. , Schiff, M. and Dinesh‐Kumar, S.P. (2002) Virus‐induced gene silencing in tomato. Plant J. 31, 777–786. 12220268 10.1046/j.1365-313x.2002.01394.x

[pbi14055-bib-0043] Livak, K.J. and Schmittgen, T.D. (2001) Analysis of relative gene expression data using real‐time quantitative PCR and the 2 (‐Delta Delta C (T)) method. Methods 25, 402–408. 11846609 10.1006/meth.2001.1262

[pbi14055-bib-0044] Lizardo, R. , Pinili, M.S. , Diaz, M. and Cumagun, C. (2022) Screening for resistance in selected tomato varieties against the root‐knot nematode *Meloidogyne incognita* in The Philippines using a molecular marker and biochemical analysis. Plants‐Basel 11, 1354. 35631779 10.3390/plants11101354 PMC9147681

[pbi14055-bib-0045] Lomsadze, A. , Burns, P.D. and Borodovsky, M. (2014) Integration of mapped RNA‐Seq reads into automatic training of eukaryotic gene finding algorithm. Nucleic Acids Res. 42, e119. 24990371 10.1093/nar/gku557 PMC4150757

[pbi14055-bib-0046] Lukasik‐Shreepaathy, E. , Slootweg, E. , Richter, H. , Goverse, A. , Cornelissen, B.J. and Takken, F.L. (2012) Dual regulatory roles of the extended N terminus for activation of the tomato Mi‐1.2 resistance protein. Mol. Plant‐Microbe Interact. 25, 1045–1057. 22512381 10.1094/MPMI-11-11-0302

[pbi14055-bib-0047] Martin, G.B. , Brommonschenkel, S.H. , Chunwongse, J. , Frary, A. , Ganal, M.W. , Spivey, R. , Wu, T. *et al*. (1993) Map‐based cloning of a protein kinase gene conferring disease resistance in tomato. Science 262, 1432–1436. 7902614 10.1126/science.7902614

[pbi14055-bib-0048] Medina Filho, H.P. and Stevens, M.A. (1980) Tomato breeding for nematode resistance: Survey of resistant varieties for horticultural characteristics and genotype of acid phosphates. Acta Hortic. 100, 383–394.

[pbi14055-bib-0049] Milligan, S.B. , Bodeau, J. , Yaghoobi, J. , Kaloshian, I. , Zabel, P. and Williamson, V.M. (1998) The root knot nematode resistance gene *Mi* from tomato is a member of the leucine zipper, nucleotide binding, leucine‐rich repeat family of plant genes. Plant Cell 10, 1307–1319. 9707531 10.1105/tpc.10.8.1307 PMC144378

[pbi14055-bib-0050] Oka, Y. , Koltai, H. , Bar‐Eyal, M. , Mor, M. , Sharon, E. , Chet, I. and Spiegel, Y. (2000) New strategies for the control of plant‐parasitic nematodes. Pest Manag. Sci. 56, 983–988.

[pbi14055-bib-0051] Osuna‐Cruz, C.M. , Paytuvi‐Gallart, A. , Di Donato, A. , Sundesha, V. , Andolfo, G. , Aiese Cigliano, R. , Sanseverino, W. *et al*. (2018) PRGdb 3.0: a comprehensive platform for prediction and analysis of plant disease resistance genes. Nucleic Acids Res. 46, D1197–D1201. 29156057 10.1093/nar/gkx1119 PMC5753367

[pbi14055-bib-0052] Peralta, I.E. and Spooner, D.M. (2005) Morphological characterization and relationships of wild tomatoes (*Solanum* L. sect. *Lycopersicon*). Monographs In Systematic Botany 104, 227–257.

[pbi14055-bib-0053] Peralta, I.E. , Knapp, S. , Spooner, D.M. and Lammers, T.G. (2005) New species of wild tomatoes (*Solanum* SECTION *Lycopersicon*: Solanaceae) from Northern Peru. Syst. Bot. 30, 424–434.

[pbi14055-bib-0054] Ploeg, A.T. (2002) Effects of selected marigold varieties on root‐knot nematodes and tomato and melon yields. Plant Dis. 86, 505–508. 30818673 10.1094/PDIS.2002.86.5.505

[pbi14055-bib-0055] Potter, S.C. , Luciani, A. , Eddy, S.R. , Park, Y. , Lopez, R. and Finn, R.D. (2018) HMMER web server: 2018 update. Nucleic Acids Res. 46, W200–W204. 29905871 10.1093/nar/gky448 PMC6030962

[pbi14055-bib-0056] Razali, R. , Bougouffa, S. , Morton, M. , Lightfoot, D.J. , Alam, I. , Essack, M. , Arold, S.T. *et al*. (2017) The genome sequence of the wild tomato *Solanum pimpinellifolium* provides insights into salinity tolerance. Front. Plant Sci. 9, 1402. 10.3389/fpls.2018.01402 PMC6186997 30349549

[pbi14055-bib-0057] Roach, M.J. , Schmidt, S.A. and Borneman, A.R. (2018) Purge Haplotigs: allelic contig reassignment for third‐gen diploid genome assemblies. BMC Bioinformatics. 19, 460. 30497373 10.1186/s12859-018-2485-7 PMC6267036

[pbi14055-bib-0058] Roberts, P.A. and Thomason, J. (1986) Variability in reproduction of isolates of *Meloidogyne incognita* and *Meloidogyne javanica* on resistant tomato genotypes. Plant Dis. 70, 547.

[pbi14055-bib-0059] Rossi, M. , Goggin, F.L. , Milligan, S.B. , Kaloshian, I. , Ullman, D.E. and Williamson, V.M. (1998) The nematode resistance gene *Mi* of tomato confers resistance against the potato aphid. Proc. Natl. Acad. Sci. USA 95, 9750–9754. 9707547 10.1073/pnas.95.17.9750 PMC21408

[pbi14055-bib-0060] Shi, Q. , Mao, Z. , Zhang, X. , Ling, J. , Lin, R. , Zhang, X. , Liu, R. *et al*. (2018) The novel secreted *Meloidogyne incognita* effector MiISE6 targets the host nucleus and facilitates parasitism in *Arabidopsis* . Front. Plant Sci. 9, 252. 29628931 10.3389/fpls.2018.00252 PMC5876317

[pbi14055-bib-0061] Smith, P.G. (1944) Embryo culture of a tomato species hybrid. Proc. Am. Soc. Hort. Sci. 44, 413–416.

[pbi14055-bib-0062] Song, B. , Marco‐Sola, S. , Moreto, M. , Johnson, L. , Buckler, E.S. and Stitzer, M.C. (2022) AnchorWave: Sensitive alignment of genomes with high sequence diversity, extensive structural polymorphism, and whole‐genome duplication. Proc. Natl. Acad. Sci. USA 119, 1. 10.1073/pnas.2113075119 PMC8740769 34934012

[pbi14055-bib-0063] Stanke, M. , Keller, O. , Gunduz, I. , Hayes, A. , Waack, S. and Morgenstern, B. (2006) AUGUSTUS: ab initio prediction of alternative transcripts. Nucleic Acids Res. 34, W435–W439. 16845043 10.1093/nar/gkl200 PMC1538822

[pbi14055-bib-0064] Steuernagel, B. , Periyannan, S.K. , Hernandez‐Pinzon, I. , Witek, K. , Rouse, M.N. , Yu, G. , Hatta, A. *et al*. (2016) Rapid cloning of disease‐resistance genes in plants using mutagenesis and sequence capture. Nat. Biotechnol. 34, 652–655. 27111722 10.1038/nbt.3543

[pbi14055-bib-0065] Tarailo‐Graovac, M. and Chen, N. (2009) Using RepeatMasker to identify repetitive elements in genomic sequences. Curr. Protoc. Bioinformatics 25, 4.10.1‐4.10.14. 10.1002/0471250953.bi0410s25 19274634

[pbi14055-bib-0066] Thorvaldsdóttir, H. , Robinson, J.T. and Mesirov, J.P. (2013) Integrative Genomics Viewer (IGV): high‐performance genomics data visualization and exploration. Brief. Bioinform. 14, 178–192. 22517427 10.1093/bib/bbs017 PMC3603213

[pbi14055-bib-0067] TomatoGenomeConsortium (2012) The tomato genome sequence provides insights into fleshy fruit evolution. Nature 485, 635–641. 22660326 10.1038/nature11119 PMC3378239

[pbi14055-bib-0068] Trapnell, C. , Roberts, A. , Goff, L. , Pertea, G. , Kim, D. , Kelley, D.R. , Pimentel, H. *et al*. (2012) Differential gene and transcript expression analysis of RNA‐seq experiments with TopHat and Cufflinks. Nat. Protoc. 7, 562–578. 22383036 10.1038/nprot.2012.016 PMC3334321

[pbi14055-bib-0069] Trudgill, D.L. and Blok, V.C. (2001) Apomictic, polyphagous root‐knot nematodes: exceptionally successful and damaging biotrophic root pathogens. Annu. Rev. Phytopathol. 39, 53–77. 11701859 10.1146/annurev.phyto.39.1.53

[pbi14055-bib-0070] Vaser, R. , Sović, I. , Nagarajan, N. and Šikić, M. (2017) Fast and accurate de novo genome assembly from long uncorrected reads. Genome Res. 27, 737–746. 28100585 10.1101/gr.214270.116 PMC5411768

[pbi14055-bib-0071] Veremis, J.C. , Heusden, V.A.W. and Roberts, P.A. (1999) Mapping a novel heat‐stable resistance to *Meloidogyne* in *Lycopersicon peruvianum* . Theor. Appl. Genet. 98, 274–280.

[pbi14055-bib-0072] Vos, P. , Simons, G. , Jesse, T. , Wijbrandi, J. , Heinen, L. , Hogers, R. , Frijters, A. *et al*. (1998) The tomato *Mi‐1* gene confers resistance to both root‐knot nematodes and potato aphids. Nat. Biotechnol. 16, 1365–1369. 9853621 10.1038/4350

[pbi14055-bib-0073] Wang, X. , Gao, L. , Jiao, C. , Stravoravdis, S. , Hosmani, P.S. , Saha, S. , Zhang, J. *et al*. (2020) Genome of *Solanum pimpinellifolium* provides insights into structural variants during tomato breeding. Nat. Commun. 11, 5817. 33199703 10.1038/s41467-020-19682-0 PMC7670462

[pbi14055-bib-0074] Wang, X.T. , Liu, Z.Q. , Sun, S. , Wu, J.X. , Li, R. , Wang, H.J. and Cui, X. (2021) SISTER OF TM3 activates *FRUITFULL1* to regulate inflorescence branching in tomato. Hortic. Res. 8, 251. 34848688 10.1038/s41438-021-00677-x PMC8633288

[pbi14055-bib-0075] Wendel, J.F. , Jackson, S.A. , Meyers, B.C. and Wing, R.A. (2016) Evolution of plant genome architecture. Genome Biol. 17, 37. 26926526 10.1186/s13059-016-0908-1 PMC4772531

[pbi14055-bib-0076] Williamson, V.M. (1998) Root‐knot nematode resistance genes in tomato and their potential for future use. Annu. Rev. Phytopathol. 36, 277–293. 15012501 10.1146/annurev.phyto.36.1.277

[pbi14055-bib-0077] Williamson, V.M. and Kumar, A. (2006) Nematode resistance in plants: the battle underground. Trends Genet. 22, 396–403. 16723170 10.1016/j.tig.2006.05.003

[pbi14055-bib-0078] Witek, K. , Jupe, F. , Witek, A.I. , Baker, D. , Clark, M.D. and Jones, J.D. (2016) Accelerated cloning of a potato late blight‐resistance gene using RenSeq and SMRT sequencing. Nat. Biotechnol. 34, 656–660. 27111721 10.1038/nbt.3540

[pbi14055-bib-0079] Wu, W. , Shen, H. and Yang, W. (2009) Sources for heat‐stable resistance to southern root‐knot nematode (*Meloidogyne incognita*) in *Solanum lycopersicum* . Agric. Sci. China 8, 697–702.

[pbi14055-bib-0080] Zhang, W. (2020) NLR‐Annotator: a tool for de novo annotation of intracellular immune receptor repertoire. Plant Physiol. 183, 418–420. 32493798 10.1104/pp.20.00525 PMC7271797

[pbi14055-bib-0081] Zhang, L. , Hu, J. , Han, X. , Li, J. , Gao, Y. , Richards, C.M. , Zhang, C. *et al*. (2019) A high‐quality apple genome assembly reveals the association of a retrotransposon and red fruit colour. Nat. Commun. 10, 1494. 30940818 10.1038/s41467-019-09518-x PMC6445120

[pbi14055-bib-0082] Zhao, J. , Sun, Q. , Quentin, M. , Ling, J. , Abad, P. , Zhang, X. , Li, Y. *et al*. (2021) A *Meloidogyne incognita* C‐type lectin effector targets plant catalases to promote parasitism. New Phytol. 232, 2124–2137. 34449897 10.1111/nph.17690

[pbi14055-bib-0083] Zheng, H. , Shi, J. , Fang, X. , Li, Y. , Vang, S. , Fan, W. , Wang, J. *et al*. (2007) FGF: a web tool for Fishing Gene Family in a whole genome database. Nucleic Acids Res. 35, W121–W125. 17584790 10.1093/nar/gkm426 PMC1933194

