# Supplementary material for: Chromosome‐scale genome assembly‐assisted identification of Mi‐9 gene in Solanum arcanum accession LA2157, conferring heat‐stable resistance to Meloidogyne incognita
Source: Plant Biotechnol J. 2023 Apr 19;21(7):1496–509. doi: 10.1111/pbi.14055 (PMC10281608; doi:10.1111/pbi.14055)
Supplement: Supplementary file 1 — Figure S1 Genome size estimate using GenomeScope. The genome size, heterozygosity and repeat content were estimated using GenomeScope. About 50 × NGS (Illumina) reads were used to count the k‐mer and export the k‐mer count histogram using jellyfish. The genome size of LA2157 was estimated to be 672.9 Mb in length using GenomeScope method. Figure S2 Whole genome alignment among S. arcanum LA2157, S. lycopersicum Heinz 1706 and S. pimpinellifolium LA2093. Grey curve connects the syntenic regions between three genomes. Numbers represent the chromosome numbers. Figure S3 Gene structure of Sarc_034200 and sequence alignment of Sarc_034200 and Mi‐1.2. (a) Gene structure and conserved domains of Sarc_034200. (b) CDS Sequence alignment of Sarc_034200 and Mi‐1.2. (c) Amino acid sequence alignment of Sarc_034200 and Mi‐1.2, the arrows represent the conserved domains. Figure S4 Phenotypes of young leaves of LA2157 plants infiltrated by TRV1 component and TRV2‐PDS component. Phenotypes of plants infiltrated by TRV1 and TRV2‐PDS, and TRV1 and TRV2‐empty infiltrated plants were set as control. (a) and (c) control plants infiltrated by TRV1 and TRV2‐empty. (b) plants of 3 weeks after infiltrated by TRV1 and TRV2‐PDS. (d) plants of 40 days after infiltrated by TRV1 and TRV2‐PDS. Figure S5 DNA and mRNA level detection of Sarc_034200 T0 generation transgenic plants. (a, b) DNA level detection of Sarc_034200 T0 generation transgenic plants. +, positive control (plasmid); −, negative control (WT plant). (c) mRNA level detection of 30 Sarc_034200 T0 generation transgenic plants. +, positive control (plasmid); −, negative control (WT plant). Figure S6 Gene relative expression level of Sarc_034200 in 30 T0 generation transgenic seedlings. RT‐qPCR was conducted to detect the gene‐relative expression level of Sarc_034200 in 30 T0‐generation transgenic plants. The ubiquitin (UBI) gene was used for normalization. The bar chart represents the mean values ± SD. The error bars represent SDs (*** [file PBI-21-1496-s002.docx]

**
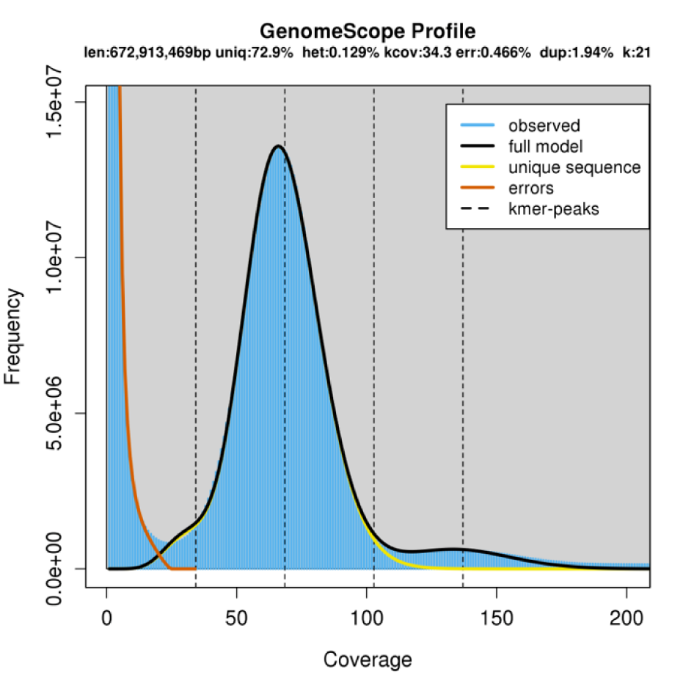
**

Figure S1 Genome size estimate using GenomeScope. The genome size, heterozygosity and repeat content were estimated using GenomeScope. About 50 x NGS (Illumina) reads were used to count the k-mer and export the k-mer count histogram using jellyfish. The genome size of LA2157 was estimated to be 672.9 Mb in length using GenomeScope method.

**
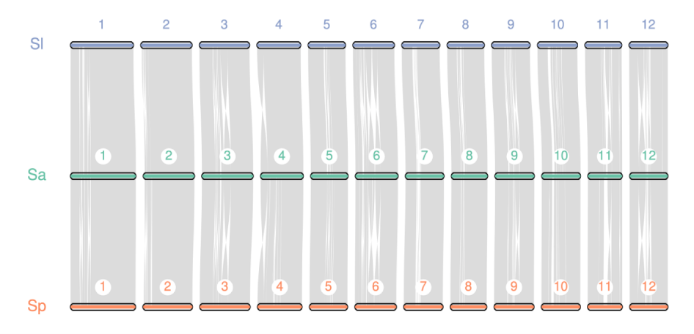
**

Figure S2 Whole genome alignment among *S. arcanum* LA2157, *S. lycopersicum* Heinz 1706 and *S. pimpinellifolium* LA2093. Gray curve connects the syntenic regions between three genomes. Numbers represent the chromosome numbers.


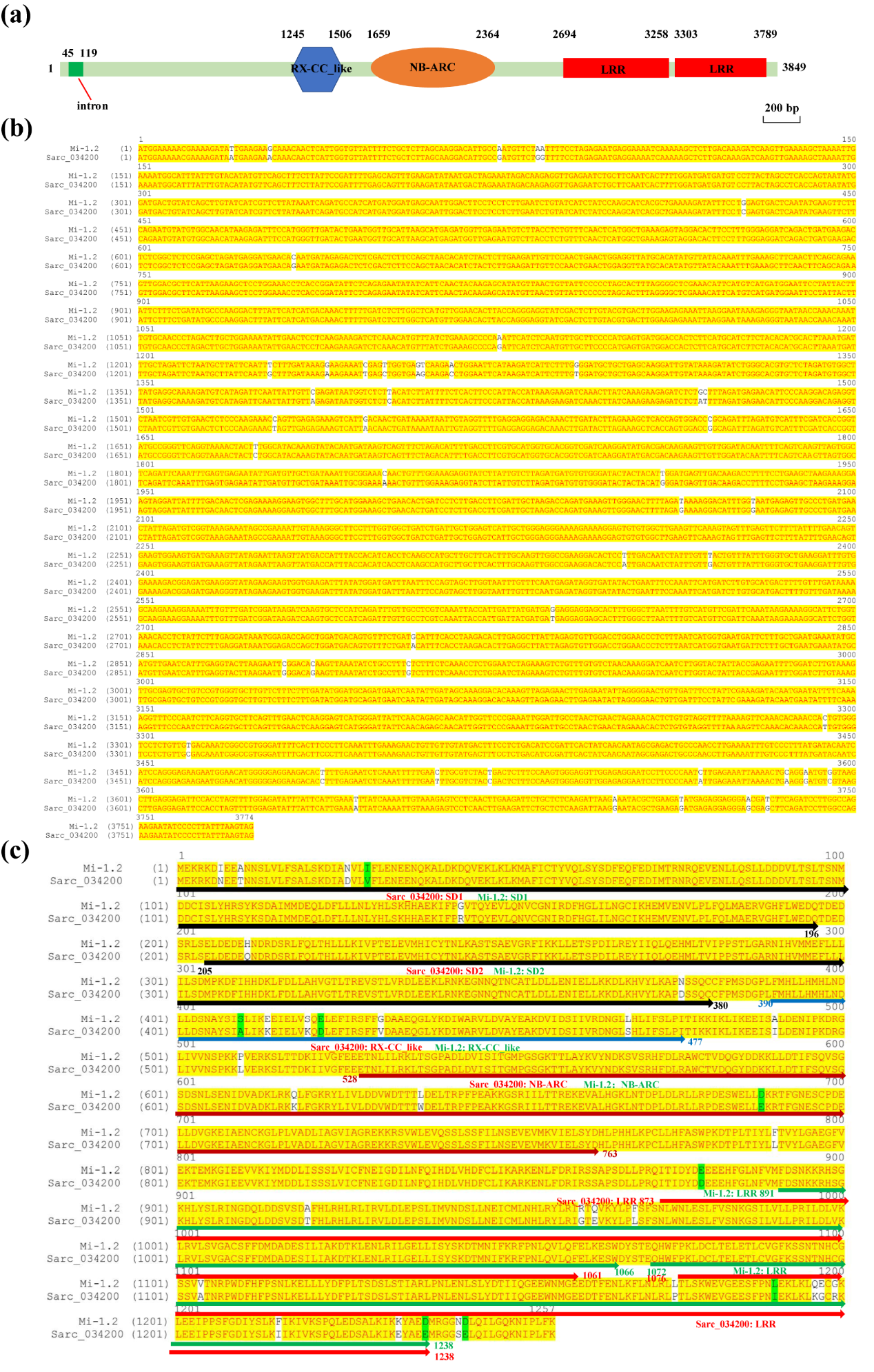


Figure S3 Gene structure of *Sarc_034200* and sequence alignment of *Sarc_034200* and *Mi-1.2*. (a) Gene structure and conserved domains of *Sarc_034200*. (b) CDS Sequence alignment of *Sarc_034200* and *Mi-1.2*. (c) Amino acid sequence alignment of *Sarc_034200* and *Mi-1.2*, the arrows represent the conserved domains.


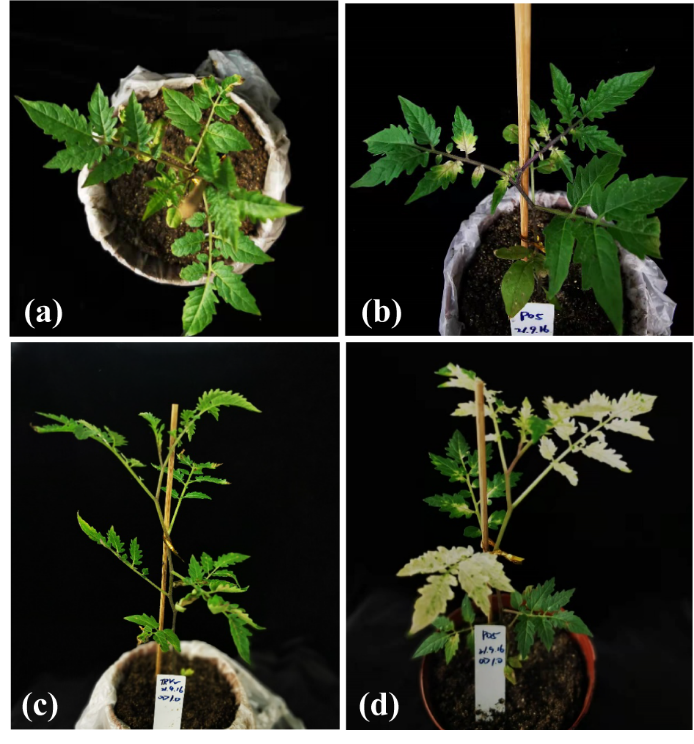


Figure S4 Phenotypes of young leaves of LA2157 plants infiltrated by TRV1 component and TRV2-*PDS* component. Phenotypes of plants infiltrated by TRV1 and TRV2-*PDS*, and TRV1 and TRV2-empty infiltrated plants were set as control. (a) and (c) control plants infiltrated by TRV1 and TRV2-empty. (b) plants of three weeks after infiltrated by TRV1 and TRV2-*PDS*. (d) plants of 40 days after infiltrated by TRV1 and TRV2-*PDS*.


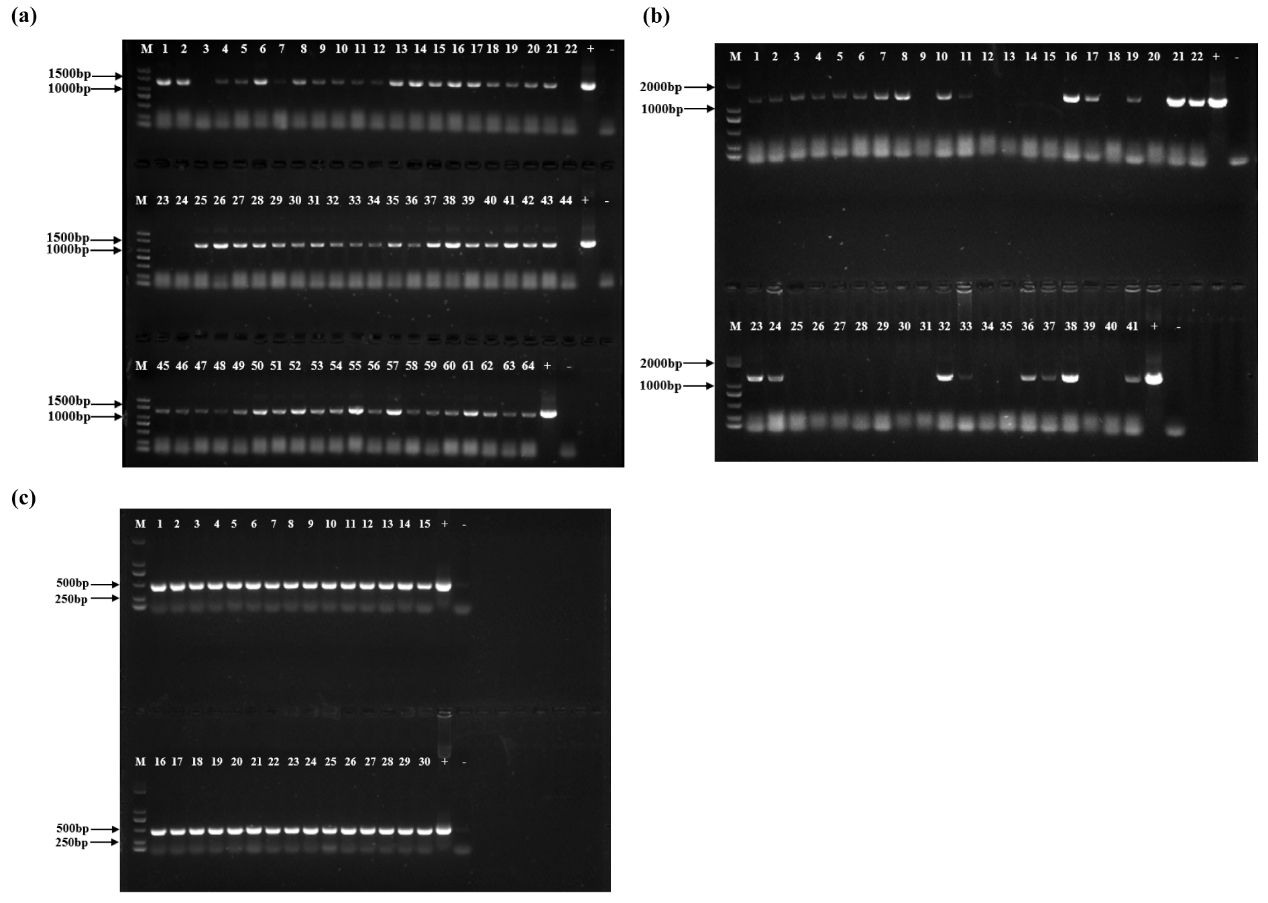


Figure S5 DNA and mRNA level detection of *Sarc_034200* T0 generation transgenic plants. (a)-(b) DNA level detection of *Sarc_034200* T0 generation transgenic plants. +, positive control (plasmid); -, negative control (WT plant). (c) mRNA level detection of 30 *Sarc_034200* T0 generation transgenic plants*.* +, positive control (plasmid); -, negative control (WT plant).


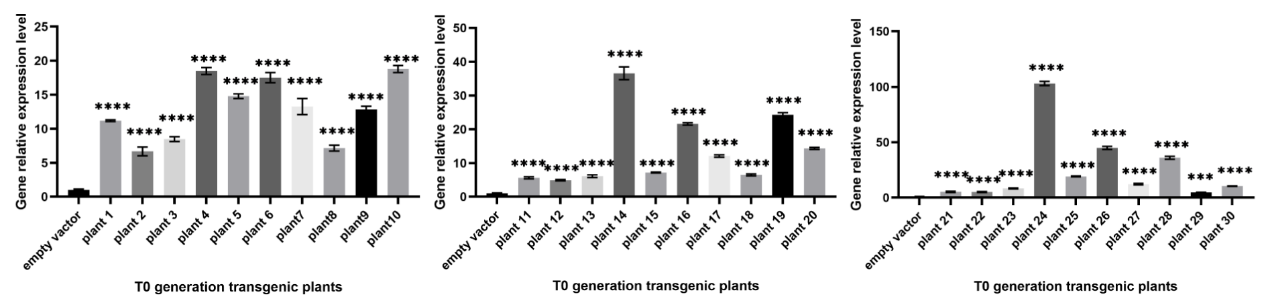


Figure S6 Gene relative expression level of *Sarc_034200* in 30 T0 generation transgenic seedings. RT-qPCR was conduct to detect the gene relative expression level of *Sarc_034200* in 30 T0 generation transgenic plants. The *ubiquitin* (*UBI*) gene was used for normalization. The bar chart represents the mean values ± SD. The error bars represent SDs (***, *P* < 0.001). Data was analyzed by one-way ANOVA followed by Dunnett’s multiple comparisons test.


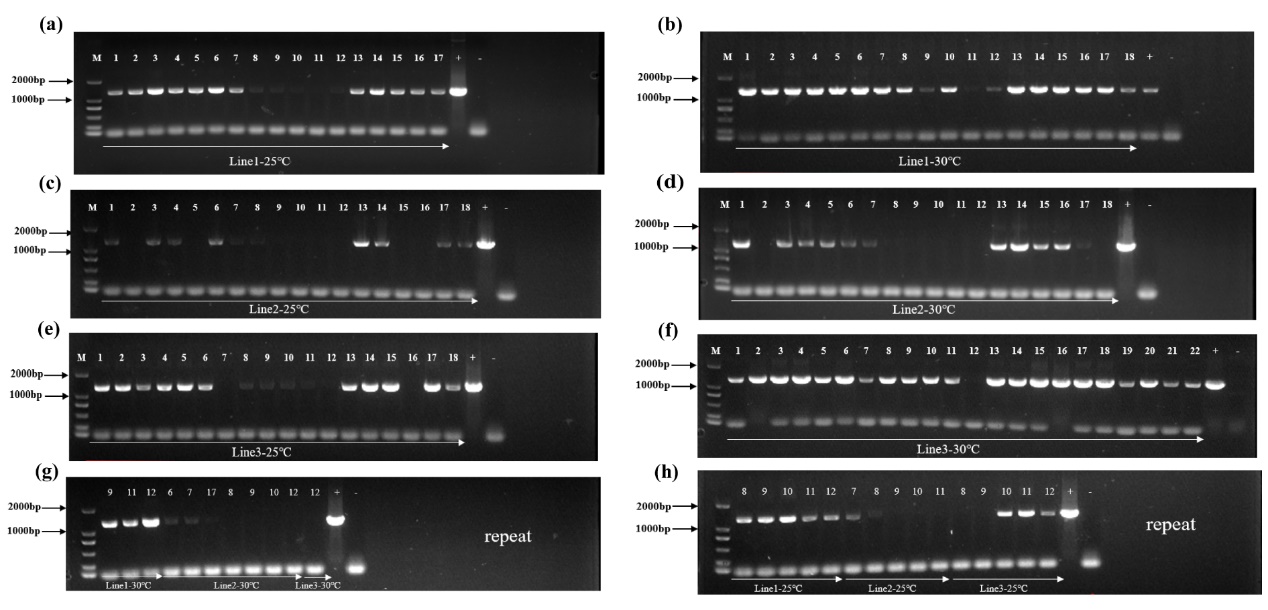


Figure S7 DNA level detection of three *Sarc_034200* T1 generation transgenic lines**.** (a), (c), (e) Detection of *Sarc_034200* with specific primers in three *Sarc_034200* T1 generation transgenic lines under the temperature of 25℃. (b), (d), (f) Detection of *Sarc_034200* with specific primers in three *Sarc_034200* T1 generation transgenic lines under the temperature of 30℃. (g) and (h) A repeat detection of the plants with no bands or weak bands. +, positive control (plasmid); -, negative control (WT plant).
