# Supplementary material for: Chromosome‐scale genome assembly‐assisted identification of Mi‐9 gene in Solanum arcanum accession LA2157, conferring heat‐stable resistance to Meloidogyne incognita
Source: Plant Biotechnol J. 2023 Apr 19;21(7):1496–509. doi: 10.1111/pbi.14055 (PMC10281608; doi:10.1111/pbi.14055)
Supplement: Supplementary file 2 — Table S1 Nanopore raw data and final assembled data of LA2157 genome. Table S2 Statistics of the LA2157 genome assembly and comparison with S. lycopersicum Heinz 1706 and S. pimpinellifolium LA2093. Table S3 The gene function annotation of LA1257. Table S4 Collinearity analysis between LA2157 and two susceptible species (Heinz 1706 and LA2093). Table S5 The genetic locus of NBS‐LRR genes in LA2157. Table S6 NBS‐LRR genes identified from the genome of LA2157, S. pimpinellifolium LA2093 and S. lycopersicum (Sly). Table S7 The gene function annotation of candidate Sarc_034200 region. Table S8 NBS‐LRR genes identified from706 kb region of LA2157, S. pimpinellifolium LA2093 and S. lycopersicum Heinz 1706. Table S9 Primes for silenced fragments of Sarc_034200 candidate genes. Table S10 qPCR primers for detection of the efficiency of gene silencing. Table S11 Primers for detection of Sarc_034200 T0 generation transgenic plants. [file PBI-21-1496-s001.zip › Supplementary materials-Table.S1-S2 S4-S6 S9-S11.pdf]

Table S1 Nanopore raw data and final assembled data of LA2157 genome

statistic for nanopore reads		statistic for assembly	
Nanopore reads	3,546,877	toatal length of contigs(Mb)	862.4
subreads length(bp)	82,902,574,641	No. of contigs	144
subreads N50(bp)	33,800	contig N50(Mb)	10.8
average subreads	23,373	toatal length of pseudomolecules (Mb)	855.68
		No. of pseudomolecules	12
		pseudomolecules N50(Mb)	69.58

Table S2 Statistics of the LA2157 genome assembly and comparison with *Sl* and *Sp*

	Sa †	*Sl*†(Hosmani et al., 2019)	*Sp*†(Wang et al., 2020)
Genome size	862,366,135	782,475,302	807,612,503
Number of contigs	144	448	500
Max length (contig)	44,760,322	26,291,688	35,406,112
N50 (contig)	10,850,275	6,007,830	10,561,219
Scaffold (HiC)	13	13	13
GC (%)	34.67	34.34	34.48
Number of coding genes	33,489	34,688	35,761
Average length (aa†)	367.31	335.04	365.6
Average number of exon	4.87	4.74	5.82
Average exon length	226.95	223.38	801.86
Average intron length	646.53	589.4	795.45
Genome completeness (BUSCO)	98.30%	97.90%	98.40%

†aa: amino acid; *Sa*: *S. arcanum* LA2157; *Sl*: *S. lycopersicum* Heinz1706; *Sp*: *S. pimpinellifolium* LA2093

Table S4 Collinearity analysis between LA2157 and two susceptible species (Heinz 1706 and LA2093)

	LA2157 and Heinz 1706			LA2157 and LA2093		
Variation type	counts	LA2157	Heinz 1706	counts	LA2157	LA2093
Syntenic regions	923	257,338,456	242,057,776	1104	286,231,217	271,284,217
Inversions	264	191,381,456	185,983,266	284	182,312,198	192,812,457
Translocations	1373	9,431,121	9,305,522	1426	9,821,234	9,709,561
Duplications (reference)	665	4,302,073	-	682	4,513,674	-
Duplications (query)	928	-	5,371,328	962	-	5,410,126
Not aligned (reference)	3102	400,180,579	-	3015	414,267,126	-
Not aligned (query)	3206	-	339,910,421	3171	-	421,021,521

Table S5 The genetic locus of NBS-LRR genes in LA2157

ch01	Sarc_000778	3359273	3364324	-	1
ch01	Sarc_001180	21500557	21506132	-	2
ch01	Sarc_001298	30006188	30006635	+	3
ch01	Sarc_001303	30739614	30740647	+	4
ch01	Sarc_002017	80918308	80919758	+	5
ch01	Sarc_002133	83389616	83390011	+	6
ch01	Sarc_002135	83394431	83397864	+	7
ch01	Sarc_002249	85425248	85430842	-	8
ch01	Sarc_002441	87588098	87593883	-	9
ch01	Sarc_002568	88907259	88910108	+	10
ch01	Sarc_002587	89202794	89205709	-	11
ch01	Sarc_002628	89645193	89648178	-	12
ch01	Sarc_002707	90509839	90515538	-	13
ch01	Sarc_002842	91772503	91776395	+	14

ch01	Sarc_003660	98541882	98545485	+	15
ch01	Sarc_003791	99649961	99654513	+	16
ch01	Sarc_003795	99672236	99676602	-	17
ch01	Sarc_003796	99679555	99683449	-	18
ch01	Sarc_003800	99697690	99698642	+	19
ch01	Sarc_003861	100262255	100262941	-	20
ch01	Sarc_004156	102472986	102476750	-	21
ch02	Sarc_028500	24284724	24286653	+	1
ch02	Sarc_028609	28805226	28808550	+	2
ch02	Sarc_028629	29259260	29260475	-	3
ch02	Sarc_028733	32210230	32222242	-	4
ch02	Sarc_028734	32228305	32229824	+	5
ch02	Sarc_028780	33138592	33147494	+	6
ch02	Sarc_028833	34457926	34462455	+	7
ch02	Sarc_028859	35073321	35077083	+	8
ch02	Sarc_029640	44348746	44350910	-	9
ch02	Sarc_029643	44371136	44373500	+	10
ch02	Sarc_029674	44598065	44599485	-	11
ch02	Sarc_029675	44601322	44604173	-	12
ch02	Sarc_030363	50312237	50317791	+	13
ch02	Sarc_030660	52589138	52591690	-	14
ch02	Sarc_031200	56832632	56836442	+	15
ch02	Sarc_031576	59772235	59773449	+	16
ch03	Sarc_012413	474855	477555	-	1
ch03	Sarc_012414	479242	483056	+	2
ch03	Sarc_012415	489604	492195	+	3
ch03	Sarc_012527	1336611	1339715	+	4
ch03	Sarc_012609	2187221	2192493	+	5
ch03	Sarc_013195	12489089	12491698	-	6
ch03	Sarc_013959	55021364	55028747	-	7
ch03	Sarc_014685	63692410	63695939	-	8
ch04	Sarc_023530	843145	845691	+	1
ch04	Sarc_023528	864343	866874	+	2
ch04	Sarc_023485	1217792	1221860	+	3
ch04	Sarc_023484	1229628	1230673	+	4
ch04	Sarc_023483	1234531	1235961	+	5
ch04	Sarc_023472	1301722	1303566	-	6
ch04	Sarc_023471	1303645	1305063	-	7
ch04	Sarc_023470	1307233	1309496	-	8
ch04	Sarc_023468	1312396	1314927	-	9
ch04	Sarc_023467	1318871	1319621	-	10
ch04	Sarc_023466	1320326	1321969	-	11
ch04	Sarc_023367	2147259	2151697	-	12

ch04	Sarc_023366	2162087	2172006	-	13
ch04	Sarc_023365	2179981	2183727	-	14
ch04	Sarc_023364	2186846	2192096	-	15
ch04	Sarc_023363	2201639	2205047	+	16
ch04	Sarc_023362	2208449	2212159	+	17
ch04	Sarc_023361	2222241	2230253	+	18
ch04	Sarc_023360	2232214	2236138	+	19
ch04	Sarc_023359	2244791	2245579	+	20
ch04	Sarc_023358	2247140	2251526	+	21
ch04	Sarc_023292	2974200	2976464	+	22
ch04	Sarc_023276	3086811	3091677	-	23
ch04	Sarc_023251	3307332	3312755	+	24
ch04	Sarc_023250	3312987	3313486	+	25
ch04	Sarc_023249	3314387	3317168	+	26
ch04	Sarc_023248	3318149	3322624	-	27
ch04	Sarc_023247	3332438	3340208	-	28
ch04	Sarc_023246	3344719	3348796	-	29
ch04	Sarc_023245	3368908	3374203	+	30
ch04	Sarc_023197	3820830	3824586	-	31
ch04	Sarc_023196	3826538	3832181	-	32
ch04	Sarc_023079	4946121	4952461	-	33
ch04	Sarc_023074	4975442	4981485	+	34
ch04	Sarc_023073	4985154	4991370	+	35
ch04	Sarc_023071	4994420	4995967	+	36
ch04	Sarc_023069	4998320	5001072	+	37
ch04	Sarc_022946	6422178	6427019	-	38
ch04	Sarc_022945	6445260	6448115	-	39
ch04	Sarc_022943	6495712	6496087	+	40
ch04	Sarc_022900	6971919	6973569	-	41
ch04	Sarc_022899	6973659	6974457	-	42
ch04	Sarc_022716	12994903	12996249	+	43
ch04	Sarc_022658	20540000	20542015	+	44
ch04	Sarc_022652	20819956	20824044	-	45
ch04	Sarc_022627	22853364	22855677	-	46
ch04	Sarc_022626	22906619	22908717	-	47
ch04	Sarc_022600	23956374	23958485	+	48
ch04	Sarc_022439	40698708	40703784	-	49
ch04	Sarc_022198	52380088	52389045	-	50
ch04	Sarc_022026	55765313	55767147	+	51
ch04	Sarc_021930	57154602	57157159	-	52
ch04	Sarc_021901	57606963	57608768	-	53
ch04	Sarc_021140	65665550	65668879	+	54
ch05	Sarc_015741	156478	161629	-	1

ch05	Sarc_015760	288650	292397	+	2
ch05	Sarc_015898	1437166	1446830	-	3
ch05	Sarc_015899	1450770	1460372	+	4
ch05	Sarc_015956	2113270	2121173	+	5
ch05	Sarc_015969	2305671	2308719	-	6
ch05	Sarc_016003	2553199	2558833	+	7
ch05	Sarc_016004	2560450	2564309	-	8
ch05	Sarc_016005	2565249	2569084	+	9
ch05	Sarc_016027	2712709	2716699	+	10
ch05	Sarc_016054	2972279	2987558	-	11
ch05	Sarc_016061	3070618	3073218	+	12
ch05	Sarc_016216	4634060	4638097	-	13
ch05	Sarc_016226	4741105	4743348	-	14
ch05	Sarc_016227	4745356	4747446	-	15
ch05	Sarc_016228	4749327	4751462	-	16
ch05	Sarc_016393	6656053	6661246	-	17
ch05	Sarc_016451	7144347	7147993	-	18
ch05	Sarc_016463	7265909	7270382	+	19
ch05	Sarc_016464	7271653	7276748	+	20
ch05	Sarc_016489	7451453	7457344	-	21
ch05	Sarc_016492	7493716	7499933	+	22
ch05	Sarc_016925	25673096	25674641	-	23
ch05	Sarc_016926	25695410	25696521	-	24
ch05	Sarc_017067	34191979	34198055	+	25
ch05	Sarc_017376	59425222	59429455	-	26
ch05	Sarc_017386	59746600	59750897	+	27
ch05	Sarc_017482	62139766	62144452	+	28
ch05	Sarc_017551	63605047	63608127	+	29
ch05	Sarc_017915	67362324	67366888	-	30
ch05	Sarc_017916	67379026	67383260	-	31
ch05	Sarc_017917	67388854	67393011	-	32
ch05	Sarc_017918	67397667	67402058	-	33
ch05	Sarc_017919	67411794	67416016	-	34
ch05	Sarc_017920	67423559	67430725	-	35
ch05	Sarc_017921	67433973	67438101	-	36
ch05	Sarc_017922	67442290	67454022	+	37
ch05	Sarc_017957	67720185	67724718	+	38
ch06	Sarc_034231	2732153	2735953	-	1
ch06	Sarc_034228	2758007	2761849	-	2
ch06	Sarc_034224	2810483	2818446	+	3
ch06	Sarc_034201	3098410	3102241	+	4
ch06	Sarc_034200	3113154	3116774	+	5
ch06	Sarc_034198	3130675	3133620	+	6

ch06	Sarc_034196	3141777	3145607	+	7
ch06	Sarc_034194	3158818	3159930	+	8
ch06	Sarc_034193	3160094	3167454	+	9
ch06	Sarc_033827	31676478	31680154	+	10
ch06	Sarc_033611	36338162	36343168	+	11
ch06	Sarc_033035	44495249	44514558	-	12
ch06	Sarc_033000	44822489	44824774	-	13
ch06	Sarc_032999	44830110	44840633	-	14
ch06	Sarc_032997	44851318	44855363	-	15
ch06	Sarc_032995	44872250	44874426	-	16
ch06	Sarc_032861	45854146	45856943	+	17
ch06	Sarc_032071	51816954	51820148	+	18
ch06	Sarc_032067	51850463	51857062	+	19
ch07	Sarc_009402	5254506	5257403	-	1
ch07	Sarc_009400	5264630	5265583	-	2
ch07	Sarc_009317	6804993	6805427	+	3
ch07	Sarc_008756	53226889	53228884	-	4
ch07	Sarc_008459	62688619	62692215	-	5
ch07	Sarc_008458	62692348	62693048	-	6
ch07	Sarc_008279	65161123	65163153	-	7
ch07	Sarc_008152	66740216	66742870	-	8
ch07	Sarc_008151	66766027	66768624	+	9
ch07	Sarc_007902	68933962	68938588	+	10
ch07	Sarc_007344	73231895	73235181	+	11
ch08	Sarc_025960	310998	313781	+	1
ch08	Sarc_025955	360112	363582	-	2
ch08	Sarc_025954	365566	368639	-	3
ch08	Sarc_025768	2098172	2102802	-	4
ch08	Sarc_025596	3876713	3884992	+	5
ch08	Sarc_024496	56747316	56750122	-	6
ch08	Sarc_024495	56750939	56751500	+	7
ch08	Sarc_024361	58375120	58378364	+	8
ch08	Sarc_024359	58387260	58390548	+	9
ch08	Sarc_024356	58414639	58417924	+	10
ch08	Sarc_024317	58743145	58747025	+	11
ch08	Sarc_023750	63708250	63714094	-	12
ch09	Sarc_004751	356064	358856	+	1
ch09	Sarc_004895	1455671	1458716	+	2
ch09	Sarc_004923	1738285	1742800	+	3
ch09	Sarc_005169	4294134	4295578	+	4
ch09	Sarc_005586	16635833	16636657	-	5
ch09	Sarc_006112	60036339	60037950	+	6
ch09	Sarc_006124	60147854	60151417	+	7

ch09	Sarc_006473	70631821	70635391	+	8
ch09	Sarc_007172	78555419	78557971	-	9
ch09	Sarc_007180	78620903	78624857	+	10
ch09	Sarc_007242	79132159	79135998	-	11
ch09	Sarc_007247	79171532	79175275	+	12
ch09	Sarc_007248	79179814	79183545	+	13
ch09	Sarc_007254	79207371	79221323	+	14
ch10	Sarc_020462	2576355	2579018	+	1
ch10	Sarc_020461	2581805	2584161	+	2
ch10	Sarc_020460	2587608	2590351	+	3
ch10	Sarc_019725	40589474	40592589	-	4
ch10	Sarc_019620	46254215	46254859	-	5
ch10	Sarc_019619	46254913	46255437	-	6
ch10	Sarc_019464	52024725	52035109	-	7
ch10	Sarc_019440	52973981	52977300	+	8
ch10	Sarc_019313	57098250	57099065	+	9
ch10	Sarc_019295	57487868	57489412	-	10
ch10	Sarc_019288	57560409	57561627	+	11
ch10	Sarc_019246	58494715	58495548	+	12
ch10	Sarc_019243	58530809	58531660	+	13
ch10	Sarc_019240	58559523	58560386	+	14
ch10	Sarc_019237	58584124	58584812	+	15
ch10	Sarc_019229	58684206	58687774	+	16
ch10	Sarc_019225	58777630	58780119	+	17
ch10	Sarc_019223	58803744	58804127	+	18
ch10	Sarc_019222	58804203	58805074	+	19
ch10	Sarc_019219	58830536	58830919	+	20
ch10	Sarc_019218	58830995	58833007	+	21
ch10	Sarc_019114	60966826	60968034	+	22
ch10	Sarc_019024	62307532	62312944	+	23
ch10	Sarc_018863	63680418	63686259	+	24
ch10	Sarc_018470	67120125	67124956	+	25
ch10	Sarc_018324	68055843	68060268	-	26
ch10	Sarc_018322	68065601	68069300	-	27
ch10	Sarc_018320	68073479	68077318	-	28
ch10	Sarc_018213	68841381	68842881	-	29
ch11	Sarc_026156	1240129	1241536	-	1
ch11	Sarc_026158	1244590	1247984	-	2
ch11	Sarc_026166	1295242	1298119	+	3
ch11	Sarc_026167	1300213	1309754	+	4
ch11	Sarc_026288	2174631	2177324	+	5
ch11	Sarc_026418	3397787	3400510	+	6
ch11	Sarc_026504	4385431	4390585	-	7

ch11	Sarc_026506	4395194	4407583	-	8
ch11	Sarc_026531	4662696	4667703	-	9
ch11	Sarc_026790	7519960	7524585	+	10
ch11	Sarc_026924	11514650	11518592	-	11
ch11	Sarc_026941	12308022	12315246	+	12
ch11	Sarc_026960	13190643	13191872	-	13
ch11	Sarc_026962	13254609	13258758	+	14
ch11	Sarc_026963	13260866	13262068	+	15
ch11	Sarc_027919	60899028	60901637	+	16
ch11	Sarc_028001	61869341	61874883	-	17
ch12	Sarc_012301	318531	319725	+	1
ch12	Sarc_012250	676237	679975	-	2
ch12	Sarc_011996	2930911	2934020	+	3
ch12	Sarc_011995	2937276	2940112	+	4
ch12	Sarc_011884	3858611	3861570	+	5
ch12	Sarc_011530	23595085	23597823	-	6
ch12	Sarc_011296	27227623	27227877	-	7
ch12	Sarc_010715	63930060	63935134	-	8
ch12	Sarc_010200	70107649	70114647	-	9
ch12	Sarc_010060	71197186	71200648	+	10
ch12	Sarc_010051	71254883	71259725	-	11
ch12	Sarc_010048	71277815	71283265	-	12
ch12	Sarc_010047	71284046	71294468	-	13
ch12	Sarc_009953	72062427	72065621	+	14

Table S6 NBS-LRR genes identified from the genome of LA2157, *S. pimpinellifolium* LA2093 and *S. lycopersicum* (*Sly*)

	LA2157	LA2093	*Sly*
NBS-LRR	215	208	207
N	77	88	81
TNL	23	18	19
TN	4	1	3
CN	42	29	32
NL	42	41	41
CNL	27	31	31

Table S9 Primes for silenced fragments of *Sarc_034200* candidate genes

Accession	Primer sequence（direction: 5’-3’）	
	Sense Primer	Antisense Primer
VIGS-*PDS*	gtgagtaaggttaccgaattcGGCACTCAACT TTATAAACCCTGAC	cgtgagctcggtaccggatccCTTCAGTT TTCTGTCAAACCATATATGT
VIGS-*Sarc_03 4201*	gtgagtaaggttaccgaattcGAAAAGGAAGT GGCTTTGCATG	cgtgagctcggtaccggatccATATTCAA CTCATAGATTGTCGTTATAGT GT
VIGS-*Sarc_03 4200*	gtgagtaaggttaccgaattcGAAGGGTATAG AAGAAGTGGTGAAGAT	cgtgagctcggtaccggatccTTAAAGA GGGTTCCAGGTCCAA
VIGS-*Sarc_03 4198*	gtgagtaaggttaccgaattcCATTTACACAA ACTCGTGCTTTCC	cgtgagctcggtaccggatccTTCTCAAA GGTGTCTTCCTCCCC
VIGS-*Sarc_03 4196*	GTGAGTAAGGTTACCgaattcTGGGT CATGTTGGAACACTTACC	cgtgagctcggtaccggatccTTGCTCAG CATCCGCAAAG

Table S10 qPCR primers for detection of the efficacy of gene silencing

Accession	Primer sequence（direction: 5'-3'）	
	Sense Primer	Antisense Primer
q-*PDS*	GATAAGTGTTAAGGACTGGATGAG	CTCTCAGGAGGATTACCATCTA
q-*Sarc_034201*	GCTGATGAGGAGCACTTCGGGC	TCACCATGATAAAAGAGTTATCC
q-*Sarc_034200*	CTTCACTTTGCAAGTTGGCCGAAG	TCTTCACCACTTCTTCTATACCCT
q-*Sarc_034198*	GTACCCGACTTACCAACTTCATGA	AGAGAGGCAATCGTCCAGCTTGTG
q-*Sarc_034196*	GCTGGTAAATTACGGAAACAACTG	AATGCCCTTTTCTCTAATAATTCC
UBI	GCCGACTACAACATCCAGAAGG	TGCAACACAGCGAGCTTAACC

Table S11 Primers for cloning *Mi-9* and detection of *Sarc_034200* T0 generation transgenic plants

Accession	Primer sequence（direction: 5'-3'）	
	Sense Primer	Antisense Primer
clone- *Sarc_034200*	cttgcatgcctgcaggtcgacGGAGACGCACATTGATAATGTCC	acggccagtgaattgttaattaaCACGCGACTTAATTGTGACACTC
Trans-DNA	GCCAAATTTAAATGACTACTTAAAG	CTAGCTCGGAGAGCCGAGAGTCTT
Trans-mRNA	GAAGGGTATAGAAGAAGTGGTGAAGAT	TTAAAGAGGGTTCCAGGTCCAA
